# Microglial activation states and their implications for Alzheimer's Disease

**DOI:** 10.1016/j.tjpad.2024.100013

**Published:** 2025-01-01

**Authors:** Zachary Valiukas, Kathy Tangalakis, Vasso Apostolopoulos, Jack Feehan

**Affiliations:** a Institute for Health and Sport, Victoria University, 70/104 Ballarat Road, Footscray VIC 3011, Australia; b First Year College, Victoria University, 70/104 Ballarat Road, Footscray VIC 3011, Australia; c School of Health and Biomedical Sciences, RMIT University, 220 3-5 Plenty Road, Bundoora VIC 3082, Australia

**Keywords:** Alzheimer's Disease, Microglia, Neuroinflammation, Amyloid Beta, Tau

## Abstract

Alzheimer's Disease (AD) is a chronic neurodegenerative disorder characterized by the accumulation of toxic amyloid-beta (Aβ) plaques and neurofibrillary tangles (NFTs) of tau protein in the brain. Microglia, key immune cells of the central nervous system, play an important role in AD development and progression, primarily through their responses to Aβ and NFTs. Initially, microglia can clear Aβ, but in AD, chronic activation overwhelms protective mechanisms, leading to sustained neuroinflammation that enhances plaque toxicity, setting off a damaging cycle that affects neurons, astrocytes, cerebral vasculature, and other microglia. Current AD treatments have been largely ineffective, though emerging immunotherapies focusing on plaque removal show promise, but often overlook the role of neuroinflammation. Activated microglia display a complex range of phenotypes that can be broadly broken into pro- or anti-inflammatory states, although this dichotomy does not describe the significant overlap between states. Aβ can strongly induce inflammatory activity, triggering the production of reactive oxygen species, inflammatory cytokines (e.g., TNF-α, IL-1β, IL-6), synapse engulfment, blood-brain barrier compromise, and impaired Aβ clearance. These processes contribute to neural tissue loss, manifesting as cognitive decline such as impaired executive function and memory. Conversely, anti-inflammatory activation exerts neuroprotective effects by suppressing inflammatory pathways and releasing neurotrophic factors that aid neuron repair and protection. Induction of anti-inflammatory states may offer a dual therapeutic approach to address both neuroinflammation and plaque accumulation in AD. This approach suggests potential strategies to modulate microglial phenotypes, aiming to restore neuroprotective functions and mitigate disease progression by simultaneously targeting inflammation and plaque pathology.

## 1 Introduction

Medical research and advancements in treatments and care have increased life expectancy worldwide, with people living longer and surviving previously deadly illnesses. As a result, age-related illnesses like dementia are becoming more prevalent [1]. The Global Burden of Disease study estimated that in 2019, approximately 57.4 million people worldwide were living with dementia, and projected to rise to 152.8 million by 2050 [2]. Evidently, there is a significant need for continued research into the etiology, treatment, and prevention of neurodegenerative diseases. Comprising 60-80 % of dementia cases, Alzheimer's Disease (AD) is the most common form of dementia, making it a critical target for research [3]. The primary pathophysiological characteristics of AD are the aggregation of amyloid-β (Aβ) plaques and neurofibrillary tangles (NFTs) of Tau protein [4]. These plaques aggregate when clearance of the aberrant proteins is inhibited, leading to gross accumulation both extra- and intracellularly [[5], [6], [7]]. Importantly, these plaques can function as seeding-points for further protein aggregation, further exacerbating the condition [4]. Additionally, they are neurotoxic, leading to neuronal death, synaptic dysfunction, and the characteristic chronic neuroinflammation seen in AD [4]. This progressive loss of healthy neural tissue causes common symptoms of dementia such as short- and long-term potentiation dysfunction, lowered inhibition, depression, and inhibited executive function [8]. Despite decades of research, much about the pathogenesis and effective treatment of AD remains unknown.

Microglia have emerged as central players in the neuroinflammatory cascade of AD pathology. As the primary immune cells within the central nervous system (CNS), these sentinels have a wide variety of roles in neuroinflammation, debris clearance, synaptic maintenance, and communicative roles amongst themselves, neurons, and astrocytes [9,10]. Microglia demonstrate similarities to peripheral macrophages in the form of activation states. Activation states can be broadly categorized as homeostatic: at rest, surveillant, examining their surroundings [11], or reactive: engaged, alert, ready to respond [12]. Furthermore, reactive microglia exhibit a fluid-like nature in their roles as inflammatory mediators, having a range of pathways involved in conversion to pro-inflammatory and anti-inflammatory phenotypes and can be simplified to the classical M1 and M2 respectively. It is worth noting that while the use of the M1 and M2 dichotomy will be used in this review for the sake of broad discussion of activation pathways, microglial responses and signals in AD, and possible therapeutics, the true nature of microglial activation is far more complex and inter-woven than this nomenclature allows, and a more comprehensive exploration of the intermediate and disease-associated microglia (DAM) [13] states is crucial in further investigations. In the early stages of AD, reactive microglia can effectively intervene in Aβ deposition through phagocytosis, barrier formation around plaques, and enzymatic degradation of Aβ [14], then ultimately return to their resting state. However, overactivation of inflammatory processes and inhibition of anti-inflammatory signaling triggers a decrease in the phagocytic and degradative capacity of microglia [12]. This results in a positive feedback loop that continually triggers microglial activation, exacerbating neuronal damage and plaque deposition [12]. Herein, we present the latest insights into microglia's involvement in Aβ-related pathologies. We discuss their activation states, their role in clearing Aβ, their interactions with other cells during disease progression, and potential strategies to shift their phenotype for therapeutic purposes.

## 2 Pathogenesis of AD

While the direct causes of AD are still unknown, the presence of Aβ and NFT plaques are the primary pathophysiological hallmarks, with several documented effects against neuronal survival. This toxicity arises from a number of mechanisms, including cell membrane disruption [15], synaptic loss [16], oxidative stress [17,18], and immune-mediated inflammation [7,9,12]. This damage leads to increased plaque buildup and inflammation, resulting in a positive feedback loop, with no effective interventions currently available. These roles in the AD disease state highlight Aβ plaques and NFTs as key targets for ongoing research and treatment development.

### 2.1 Formation of Aβ

**Fig. 1:**
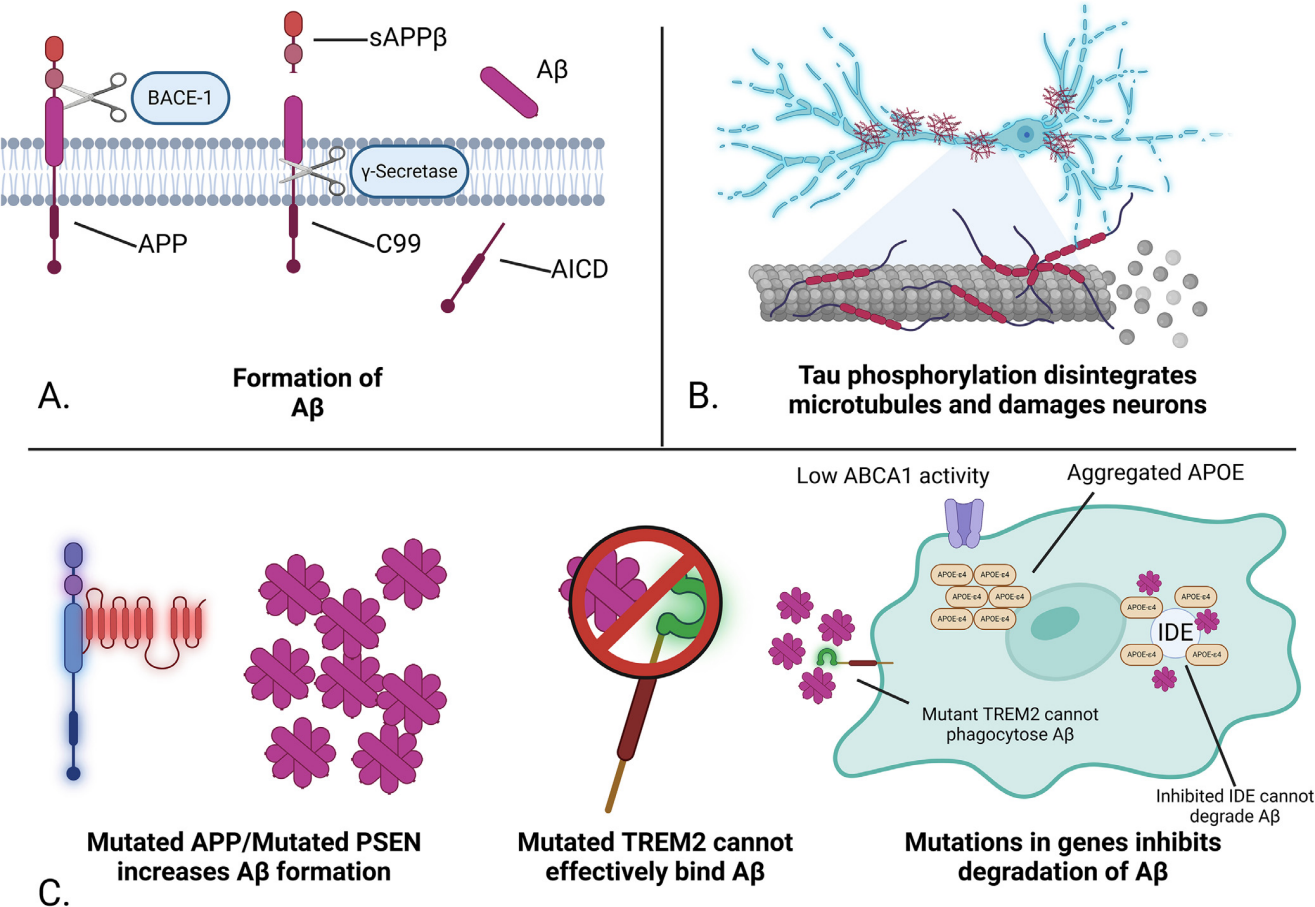
**A.** Formation of Aβ, BACE-1 cleaves APP generating sAPPβ and C99, then cleaved by γ-secretase to generate free Aβ and AICD. **B.** Microtubule destabilization NFT formation collapses microtubules in neurons inhibiting cargo transport and destroying cellular structure. **C.** The effects of specific gene mutations in AD have significant effects on Aβ processing. Mutated APP and mutated PSEN cause greatly increased Aβ formation. Mutations in TREM2 significantly inhibit microglial binding to Aβ, leaving aggregates in the extracellular space. The APOE-ε4 isoform inhibits lipid processing by ABCA1 and the activity of IDE, one of the primary enzymes used for Aβ degradation.

Aβ is an insoluble 36-43 amino acid peptide that is generated through a cascade of enzymatic cleavage. Amyloid precursor protein (APP) is a membrane-bound protein first cleaved by the beta-site amyloid precursor protein cleaving enzyme 1 (BACE1) [19] leading to the generation of soluble amyloid precursor protein β (sAPPβ) and the integral fragment C99 [20]. C99 is then cleaved by γ-secretase, generating amyloid precursor protein intracellular domain (AICD) and free Aβ peptide [21] (Fig. 1). Aβ fibrils and oligomers can also seed the formation of further plaques through several mechanisms like the apolipoprotein E-epsilon 4 isotype (APOE-ε4), advanced glycation end-product (AGEs) modification, as well as dysfunctional microglial clearance [[22], [23], [24]]. Notably, while genetic driven Aβ generation is typically followed by NFT aggregation [25,26], tauopathies may not trigger Aβ formation [4].

### 2.2 Tau phosphorylation

NFTs are large aggregates of hyperphosphorylated microtubule associated protein Tau (Tau) that have disengaged from neuronal microtubules and formed large, disordered structures. The phosphorylation of Tau can occur at a possible 85 known sites, 28 exclusively in AD brains [27], and directly reduces its affinity for tubulin and microtubule polymerization [28]. However, this plays a major role in healthy axonal cargo transport [29,30], with recent evidence suggesting that nuclear localized phosphorylated Tau compacts chromatin for DNA and RNA protection [31]. Hyperphosphorylation is characterized by a 3- to 4-fold increase in phosphorylated sites compared to controls [32], causing axonal collapse due to loss of microtubule stability (Fig. 1) [29].

### 2.3 The dual plaque hypothesis

AD is an incredibly complex disease with several areas of CNS dysfunction and damage. Historically, efforts have focused on the Amyloid-hypothesis – the epicenter of AD development and progression is the formation of Aβ plaques – with much evidence pointing in this direction. However, discontent with ineffective anti-Aβ therapies has driven attention away from Aβ and towards NFTs, having better correlation with dementia symptoms and severity than Aβ [[33], [34], [35]]. A more synergistic relationship between Aβ and NFTs may better describe AD pathology, with toxicity and signaling between the two triggering inflammation and further deposition of each other [36,37]. This interplay has led to a deeper understanding of the role of these plaques in AD progression. Despite their neurotoxic effects, there is evidence for both proteins having physiologically healthy roles in very low concentrations. Studies in mice have shown Aβ has an array of protective functions, including anti-microbial, anti-tumor, micro-hemorrhage sealing, and traumatic brain injury repair properties [38]. Additionally, Aβ may also enhance hippocampal synaptic plasticity, short-term, and long-term potentiation at low picomolar concentrations [39,40].

## 3 Risk factors for AD

There are a wide range of potential risk factors for AD, both genetic and environmental. Lifestyle factors such as smoking, sedentary behavior, drug and alcohol use, along with comorbid disease such as diabetes, obesity and infection also play significant roles in increasing the risk [41,42]. Different levels of risk across racial and ethnic groups may also be observed, where Black people (including residents of the United States, the Caribbean islands, and people across Africa) were at greater risk of AD when compared with Asian, Hispanic, and White people [2,[43], [44], [45]], however, the underrepresentation of minority groups across research studies means more data is required for comprehensive analysis of these differences. A growing area of research also focuses on the impact of gastrointestinal health and the body's microbiome, with evidence suggesting CNS inflammation may be reduced by diets rich in fiber, which boost the production of anti-inflammatory short-chain fatty acids [[46], [47], [48], [49], [50]]. Peripheral inflammation and immunosenescence can also trigger inflammatory responses and reduced hippocampal plasticity in aged mice [[51], [52], [53], [54]]. There are also specific genetic factors that increase the risk of AD manifestation. The most observed genetic factors for AD-onset are the APOE-ε4 isotype, and mutations in the triggering receptor expressed on myeloid cells 2 (TREM2), APP, presenilin-1 (PSEN1) and presenilin-2 (PSEN2) genes [[55], [56], [57]].

### 3.1 APOE-ε4

The *APOE-ε4* genotype is a strong risk factor for AD [23,[58], [59], [60]]. APOE is a significant regulator of lipid metabolism within the brain, and is primarily expressed by astrocytes, though highly expressed by microglia in AD [61]. APOE is typically processed by the ATP-binding cassette transporter ABCA1 (ABCA1), though the lipid-poor APOE-ε4 more readily forms aggregations extra- and intracellularly, particularly in acidic endosomes [62]. This inhibits its lipidation and processing by ABCA1 [60]. APOE has been found to have isoform-dependent effects, with APOE-ε3 being typical, APOE-ε2 having some protective effects against AD [63], and APOE-ε4 causing greater risk of AD. Additionally, the ε4 genotype may be expressed in varying proportions amongst different racial and ethnic groups, where Black people more commonly have at least one ε4 allele, and Hispanic people have a greater proportion of homozygous and heterozygous ε2 alleles compared with other groups [[43], [44], [45]]. A major component of this increased risk is the effect of APOE-ε4 on the proteolytic degradation of Aβ, both soluble and oligomeric. APOE is essential for efficient intracellular degradation of soluble Aβ (sAβ) by microglia, with *APOE*^-/-^ microglia almost completely impaired, and the poorly lipidated APOE-ε4 having limited capacity to degrade Aβ [64]. In addition, hypo-lipidated APOE inhibits insulin degrading enzyme's (IDE) ability to degrade Aβ [64] and a recent report highlighted the upregulation of the acyl-CoA synthetase long-chain family member 1 (ACSL1) lipid-processing enzyme in *APOE-ε4/ε4* microglia. ACSL1 is a key part of lipid droplet formation – lipid storage organelles involved in anti-microbial defense and lipid metabolism [65] – and may be a source of overabundant lipids found in microglia and neurons in AD [66]. Furthermore, a study comparing brain tissue from homozygous APOE-ε4 and -ε3 patients observed altered expression in genes associated with microglial activation (e.g., *SALL1, FSCN1, DNMT1*) in ε4/ε4 that results in a slower and weaker response to Aβ [67]. Another study examined the effects of APOE-ε3 and -ε4 expression in *APP*/*Trem2^-/-^* transgenic mice and observed several changes. Firstly, deletion of *Trem2* was correlated with increased plaque growth. Secondly, deletion of *Trem2* reduced the expression of *Apoe* mRNA in the -ε4 mice, but not in the -ε3 mice [68]. Another mechanism of APOE-ε4’s influence on Aβ deposition is through interactions with the blood brain barrier (BBB). Studies in mice models of AD demonstrated that the -ε4 isoform significantly increased levels of Aβ within cerebral arterioles compared with -ε3 [69], and a greater volume of APOE-ε4 present in these vascular plaques compared to the brain parenchyma [70,71]. In addition to these effects on Aβ transport and aggregation, post mortem studies using brain tissue of patients with AD revealed significantly increased rate of pericyte and perivascular breakdown in APOE-ε4 samples when compared with -ε3 and non-AD control samples, accompanied by increases in the inflammatory cytokine CypA and the matrix metalloprotease-9 (MMP-9) [72]. These results all show strong isoform-dependent effects on microglial responses to Aβ pathology and contribute to the key role of APOE-ε4 in the propagation of Aβ.

### 3.2 ABCA1

ABCA1 is a key transporter molecule within the CNS that facilitates the transport of intracellular cholesterol from endosomes into APOE to form APOE-HDL [73]. This process involves recycling of ABCA1 between endosomal compartments and the plasma membrane and is facilitated by the ADP-ribosylation factor 6 (ARF6). Significantly upregulated in the hippocampus of AD brains [74], increased ARF6 activity results in lower movement to the cellular membrane and greater lysosomal degradation of ABCA1 [75]. Following this, APOE lipidation is significantly reduced, leading to a direct increase in Aβ formation and deposition [76,77].

### 3.3 TREM2

TREM2 is a transmembrane protein capable of binding both glycoproteins and lipids [78]. Mutations leading to deficiency or altered protein reduce the Aβ phagocytic capability of affected microglia and more wide-spread and less compact plaques [79,80]. Microglia in AD brains express TREM2 in greater numbers, with strong affinity for oligomeric Aβ [13,56]. Additionally, TREM2 is a crucial part of microgliosis – the recruitment and proliferation of microglia – in the presence of Aβ, via its strong binding affinity for lipids on the surfaces of neurons and other glial cells damaged by Aβ [81]. The R47H variant is a TREM2 mutation that is strongly associated with increased risk of AD. R47H mutations are unable to effectively bind with phospholipid ligands [82]. One study indicated that this mutation causes conformational changes in the phospholipid binding site [83]. This dysfunction results in significantly impeded barrier formation by microglia around plaques, enhancing the spread and toxicity of Aβ [84]. In addition to its membrane bound form, soluble TREM2 (sTREM2) is found within the cerebrospinal fluid, with increasing levels of sTREM2 present in AD [9]. sTREM2 demonstrates high affinity for Aβ oligomers and APOE, acting as a putative marker for microglial intervention [6,84].

### 3.4 APP

APP is a regulator of synaptic formation and plasticity [85,86], antimicrobial activity [87] and iron transport [88]. Mutations in the amino- and/or carboxy-terminal points of the Aβ sequence (e.g. *APP*692, *APP*717) cause an increase in cleavage of APP by β- and γ-secretase [89]. Interestingly, APP has other products with neurodegenerative effects independent of Aβ. APP is a substrate of caspases-3, -6, -8 and -9, with cleavage by caspase-8 and -9 resulting in toxic fragments C31 and Jcasp [90,91]. The apoptotic effects of C31 and Aβ are dependent on the presence of suitable cleavage site on APP [92].

### 3.5 PSEN

PSEN1 and PSEN2 are protein subunits of the γ-secretase complex with approximately 200 pathogenic mutations that contribute to AD pathology [93]. These mutations generate instability in the substrate-protease complex, leading to greater production of the more toxic and aggregate prone Aβ_42_ [94]. There is also evidence suggesting *PSEN* mutations could generate pathological conditions and changes in mitochondrial function, leading to the characteristic oxidative stress and inflammation of AD [95].

## 4 Microglia activation states

The complex and inter-connected pathways of microglial activation and signaling gives rise to a unique and dynamic cell capable of quickly responding to a stimulus, then returning to rest. The idea of a pro- and anti-inflammatory dichotomy is outdated and does not adequately convey the true nuances of microglial activation, with *in vivo* studies typically yielding results both within and outside of these states. However, these classifications still hold merit as a broad descriptor of functions and responses, and will be used here to simplify the pathways and relationships between microglia, their stimuli, and their neighboring cells [14]. For more comprehensive analysis of intermediary and overlapping states, we recommend these papers by Keren-Shaul et al. [13] and Gerrits et al. [14].

### 4.1 Pro-inflammatory Microglia/M1

Pro-inflammatory microglia typically arise from interaction with interferon-gamma (IFN-γ) and lipopolysaccharide (LPS) [12]. After induction, M1 microglia initiate the release of inflammatory cytokines and chemokines including tumor necrosis factor-alpha (TNF-α), interleukin (IL)-1β, -6, -12, and CC chemokine ligand (CCL) 2 [96]. Furthermore, M1 microglia express major histocompatibility complex-II (MHC-II), integrins CD11b and CD11c, CD36, 45, 47, and Fc receptors (FcR) [96]. M1 microglia also express nicotinamide adenine dinucleotide phosphate (NADPH) oxidase and inducible nitric oxide synthase (iNOS), which produce reactive oxygen species (ROS) and nitric oxide (NO) [12]. These factors contribute to an inflammatory response capable of moving to sites of infection or disease, binding to and degrading pathogens. Importantly, these functions are necessary for homeostatic maintenance and are effective when used in a controlled manner. However, in the chronic neuroinflammation of AD, continuous expression of these signals and the resulting phenotypic changes result in loss of phagocytic function and damage to the surrounding environment, including neurons, astrocytes and cerebral capillaries, triggering oxidative stress, inflammatory cascades, and apoptosis [97,98]. Additionally, chronically activated pro-inflammatory microglia demonstrate significantly reduced Aβ uptake and degradation, further contributing to the inflammation state [64].

### 4.2 Anti-inflammatory Microglia/M2

Anti-inflammatory microglia engage in neuroprotective and controlled responses which aim to repair the local environment in the CNS. The M2 state is induced by anti-inflammatory cytokines like IL-4, -10, and -13 [99], and leads to release of anti-inflammatory cytokines such as transforming growth factor-beta (TGF-β), IL-10, insulin-like growth factor-1, fibroblast growth factor, colony stimulating factor-1 (CSF-1), pro-survival factor progranulin and the mannose receptor (CD206) [96], and promotes expression of the M1 inhibitor CD200 in other cells. In early stages of AD, M2 microglia are present and can uptake and degrade Aβ [100]. While M2 microglia can limit Aβ deposition and prevent toxicity, they are eventually overwhelmed and are unable to reduce the growing plaque volume [[101], [102], [103]]. Aβ aggregation and chronic inflammatory signaling induces internalized aggregation and external toxicity of plaques, resulting in the chronic inflammation loop seen in AD, exacerbated by a loss of M2 signals like CD200/CD200R interactions, and anti-inflammatory cytokines from neurons and astrocytes [99,104,105]. Protective pathways like PPARγ, STAT6, and AMPK/PGC-1α rely on anti-inflammatory signaling but are overpowered by factors such as aging [24], genetic mutations or pathogenic isoforms of genes (*APOE-ε4, TREM2, APP, PSEN*) [55], oxidative stress and injury from local cells (i.e., IFN-γ, TNF-α, IL-1β) [99], and inhibition of key signals and transcription factors [96,99]. Ultimately, M1 overactivation leads to conditions that strongly inhibit M2 signaling, resulting in a loss of phagocytic and degradative capacity, functional communication between cells, and unrestrained inflammation [99].

### 4.3 Disease-associated microglia

Though useful in broadly describing the induction and activity of microglia, the M1/M2 dichotomy fails to adequately describe the true complexity of microglial activity. Newer research supports a more fluid state, with many different phenotypes present, as well as ever-changing activation state due to local conditions. Within AD, a unique sub-group of microglia known as disease-associated microglia (DAMs) have been described and observed congregating around and phagocytosing Aβ plaques [13]. The conversion of homeostatic microglia to a DAM phenotype was shown to be a two-stage process. First, a TREM2-independent process results in upregulation of genes associated with AD progression (*APOE, β2m*), and TYRO protein tyrosine kinase-binding protein (TYROBP), an adapter protein that forms a signaling complex with TREM2. In addition, genes associated with microglial homeostasis (*CX3CR1, P2RY12/P2RY13*) [57] were downregulated. The second step of differentiation only occurred in TREM2^+^ microglia, and significantly upregulated TREM2 expression, as well as genes associated with lipid metabolism (*LPL*), proliferation (*CSF1*) and phagocytosis (*CD68*) [13]. Further, studies identified novel sub-groups of microglia present in AD pathology in mice [14]. These groups, termed AD1 and AD2, demonstrated distinct phenotypes, with AD1 bearing many similarities to DAMs, with significant upregulation of genes involved in migration and phagocytosis [14]. This corroborates earlier findings that demonstrated specific AD-risk genes influence microglial responses to Aβ, but not Tau pathologies [18]. Interestingly, microglia were converted to the AD1 phenotype in the presence of only Aβ, with the presence of Tau generating both AD1 and AD2 populations [14]. Furthermore, there was no identified conversion between the two. Recently, two reports investigated the role of APOE-ε4 as a modifier of DAM responses in AD and further highlighted the damaging effects of the isoform [106,107]. Yin et al., investigated the function and responses of APOE-ε4^+^ DAMs, and upregulation of Integrin beta-8 (*ITGB8*), an upstream regulator of TGFβ signaling in the brains of heterozygous ε3/ε4 male AD patients. Interestingly, upregulation of other risk genes associated with AD was observed in female patients, including *APP, CD33,* Interferon-alpha/beta receptor alpha chain (*IFNAR1),* and *ABCA7* [106]*.* Additionally, high expression of the homeostatic transcription factor PU.1 was observed in APOE-^ε4+^ microglia, and was associated with increased SMAD3, a downstream adaptor of TGFβ in the brains of female AD patients heterozygous for APOE-ε3 and -ε4. These findings show interesting sex-based differences in the effects of APOE-ε4 on microglial function and responses, and demonstrate a unique pathway for -ε4 driven progression of AD by reducing microglial protective function and enhancing plaque toxicity [106,107]. Deeper investigation of DAMs could help identify common genes amongst individuals with AD and build a more complete understanding of pathways, responses to stimuli and associated activities like lipid metabolism and inflammation [108], as well as possible areas for controlling over-zealous inflammatory activation [12,96,99,109].

### 4.4 M1 to M2 pathways

**Fig. 2:**
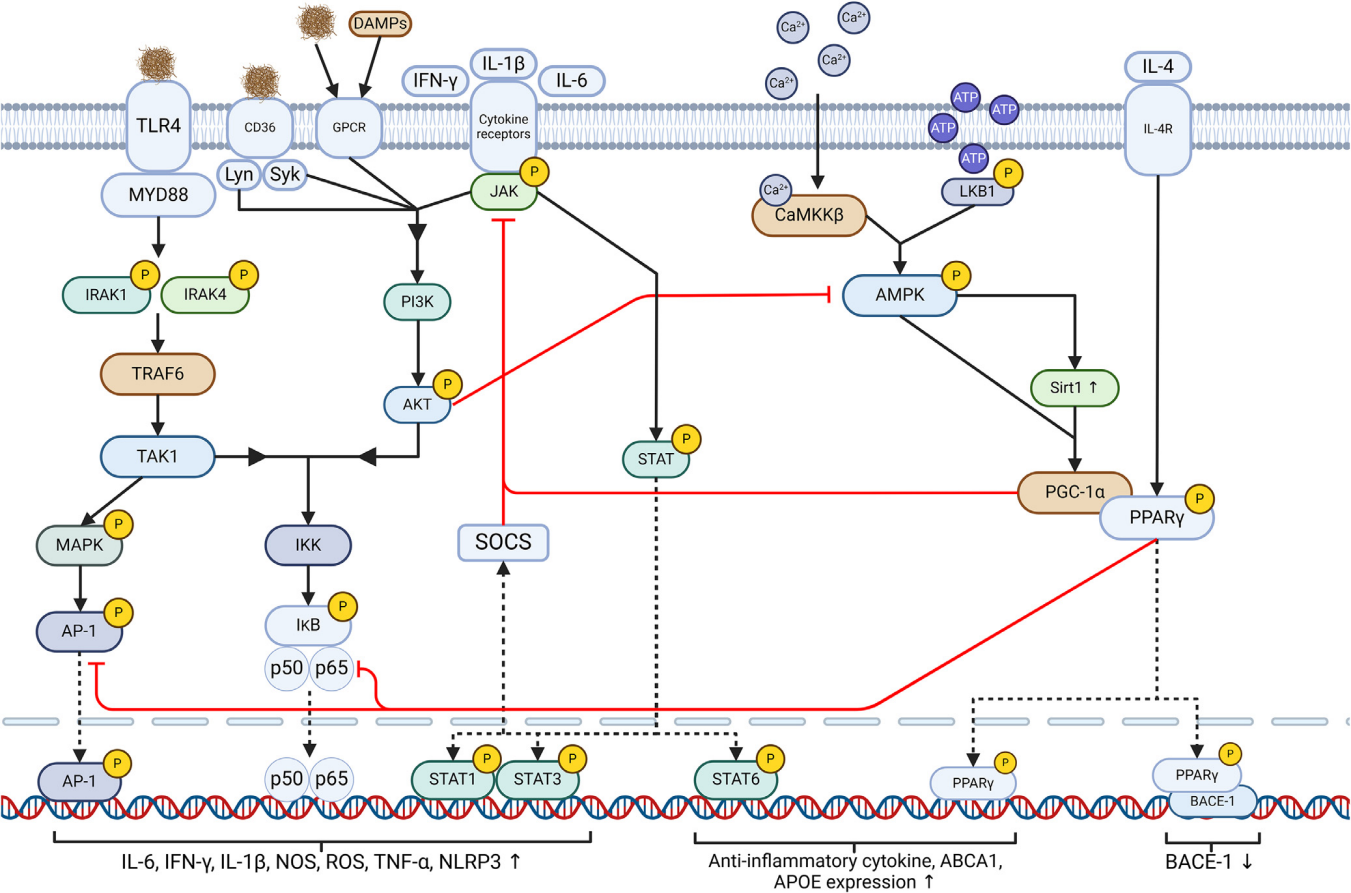
Important pathways in Microglial M1/M2 activation Inflammatory signals like insoluble Aβ associate with TLR4 and MYD88, triggering autophosphorylation of IRAK, triggering TRAF6. TRAF6 activates TAK1, triggering AP-1 via MAPK, and NF-κB by activating IKKs. Inflammatory cytokines trigger JAK/STAT pathways, with STAT1/3 activating inflammatory gene transcription and SOCS activation, and STAT6 activating anti-inflammatory transcription. RTK/GPCR and JAK activation triggers PI3K activation, which then phosphorylates AKT triggering NF-κB pathways and inhibits AMPK activation. CaMKKβ and LKB1 activation phosphorylates AMPK, triggering Sirt1 increase, and both can activate PGC-1α. PGC-1α inhibits JAK, NF-κB and AP-1 activation. PGC-1α and IL-4 activate PPARγ, increasing anti-inflammatory products. PPARγ inhibits BACE-1 and increases ABCA1/APOE, reducing Aβ generation and increasing its uptake and degradation.

With DAMs expressing many similarities to M1 activated microglia, an understanding of the complex and overlapping signals that trigger phenotypic switching may aid in the development and refinement of microglia-targeted treatments for AD. This section will briefly describe some key pathways involved in M1 → M2 switching and their relation to AD (Fig. 2**)**, with interventions covered in a later section. The full scope of M1/M2 pathways is too complex for this review, so we recommend these reviews by Guo et al. [99] and Darwish et al. [96] for more information.

#### 4.4.1 TLR4

Toll-like receptor 4 (TLR4) is a member of the pattern recognition receptor (PRR) family and is associated with inflammatory responses and strongly implicated in microglial responses to Aβ [110]. Aβ-activated TLR4 associates with myeloid differentiation factor 88 (MyD88), which triggers autophosphorylation of interleukin-1 receptor-associated kinase (IRAK) -1 and -4. IRAK-1/-4 then interact with tumor necrosis factor receptor associated factor-6 (TRAF6) to activate transforming growth factor-β-activated kinase 1 (TAK1) [98]. This triggers nuclear factor kappa-B (NF-κB) and mitogen-activated protein kinase (MAPK) pathway activation inducing transcription of pro-inflammatory genes [98,111]. This activation by Aβ starts a cascade of signals that triggers inflammatory pathways to quickly respond to and clear plaques. Importantly, TLR4 works with the coreceptor CD14 to uptake degradation resistant fibrillar Aβ (fAβ) [112], leaving plaques intact intra- and extracellularly and reactivating TLR4 and internal signaling. Additionally, TLR4, TLR2 and CD14 are required for downstream p38 MAPK activation, microglia deficient in these receptors showing reduced p38 activation [110].

#### 4.4.2 NF-κB

NF-κB is a crucial transcription factor for M1 activation, with inhibition of its p65/p50 subunits promoting M2 activation [113]. Constantly present within the cytoplasm, NF-κB inhibitors (IκB) work to prevent activation [111]. IκB are phosphorylated and degraded by the TAK1 activated IκB kinase (IKK), allowing p50/p65 ingress to the nucleus and triggering inflammatory gene transcription [97].

#### 4.4.3 MAPK

MAPK activation by TAK1 induces the phosphorylation and activation of the transcription factor activator protein-1 (AP-1) [111]. MAPK itself is composed of several signal molecules with further downstream pathways, including the c-Jun NH2-terminal kinases (JNKs), p38 MAPK and the p42/p44 extracellular signal-regulated kinase (ERK), with inhibition of this pathway subduing M1 activation [114]. Specific inhibition of p38 directly reduces fAβ-induced ROS production and phagocytosis of fAβ, thereby reducing inflammatory activation [110].

#### 4.4.4 AMP-activated protein kinase

Adenosine monophosphate-activated protein kinase (AMPK) is an enzyme involved in energy maintenance [115] and is phosphorylated by the calcium/calmodulin-dependent protein kinase kinase-β (CaMKKβ) after Ca^2+^ influx during neuroinflammation [116,117]. Activated AMPK can stimulate the peroxisome proliferator-activated receptor γ coactivator 1α (PGC-1α), a key regulator in mitochondrial biogenesis and metabolism [118]. In addition, AMPK increases NAD^+^ volumes, increasing Sirtuin 1 (Sirt1) activation, which deacetylates and activates PGC-1α [118]. PGC-1α also stimulates the peroxisome proliferator-activated receptor γ (PPARγ), an important mediator of M2 signaling [119,120]. Activation of AMPK/CaMKKβ follows microglial activation and works to control the subsequent immune response. These pathways are indirectly inhibited by PI3K/AKT signaling within DAMs [13,121], showing a link between Aβ as a trigger for inflammation and suppressor of anti-inflammatory responses.

#### 4.4.5 PPARγ

PPARγ is nuclear receptor suite of ligand-inducible transcription factors and triggers M2 expression after stimulation with IL-4 [99]. Furthermore, PPARγ has protective effects, able to antagonize inflammatory factors AP-1, NF-κB, and the signal transducer and activator of transcriptional signals 1 (STAT1) [122]. Further, studies in APP/PS1 mice demonstrated significantly reduced Aβ volume and improved cognitive function, following treatment with PPARγ agonists pioglitazone [123] and rosiglitazone [124] by increasing ABCA1 and APOE expression. Additionally, the BACE-1 gene contains a PPARγ specific promoter region that suppresses BACE-1 expression and results in lowered Aβ formation and deposition [125].

#### 4.4.6 Janus Kinase/STAT

Janus Kinase (JAK) and STAT signaling are a combined pathway of transcription regulators. JAK activation phosphorylates STATs, causing their translocation to the nucleus and transcription of relevant genes, including the negative feedback regulator suppressors of cytokine signaling (SOCS). SOCS proteins inhibit JAK activation, controlling STAT family activation and subsequently gene transcription [126]. STAT1 and STAT3 induce pro-inflammatory cytokine and chemokine production, while STAT 6 may promote M2 activation [99]. Additionally, phosphorylated JAK is necessary for the activation of the PI3K/AKT signal pathway [127]. While JAK/STAT signaling can shift microglia to M1 or M2, chronic inflammation signals in AD like IFN-γ and IL-6 continuously activate STAT1/STAT3 pathways leading to increases of inflammatory cytokines like IFN-γ, IL-1β, and IL-6 increasing NF-κB, p38 and JAK/STAT activation [128,129].

#### 4.4.7 PI3K/AKT

Receptor tyrosine kinases (RTK) like Lyn and Syk, and GPCR activation cause the activation of phosphoinositide 3-kinase (PI3K), which then phosphorylates AKT/Protein kinase B (PKB). NF-κB is a downstream factor of PI3K/AKT signaling, and indirectly prevents AMPK activation by phosphorylating AMPK, inhibiting the activity of another AMPK activator, liver kinase B1 (LKB1) [99]. This results in significant inhibition of regulatory pathways that control inflammatory responses such as PGC-1α and Sirt1 activation, decreasing PPARγ activity. Importantly, both RTK and GPCR activation can occur from interactions with Aβ, and damage-associated molecule patterns (DAMPs) released by other cells during inflammation [130]. The wide variety of pathways that can trigger M1 → M2 switching highlights a potential area for pharmacologic intervention. Chronic stimulation from Aβ and loss of degradative function play a crucial role in starting this cycle, so interventions that can rescue microglia from a chronically inflamed state by inducing anti-inflammatory responses may be able to elicit neuroprotective effects and stimulate Aβ clearance.

## 5 Microglia and Aβ

### 5.1 Receptor mediated interaction with Aβ

**Fig. 3:**
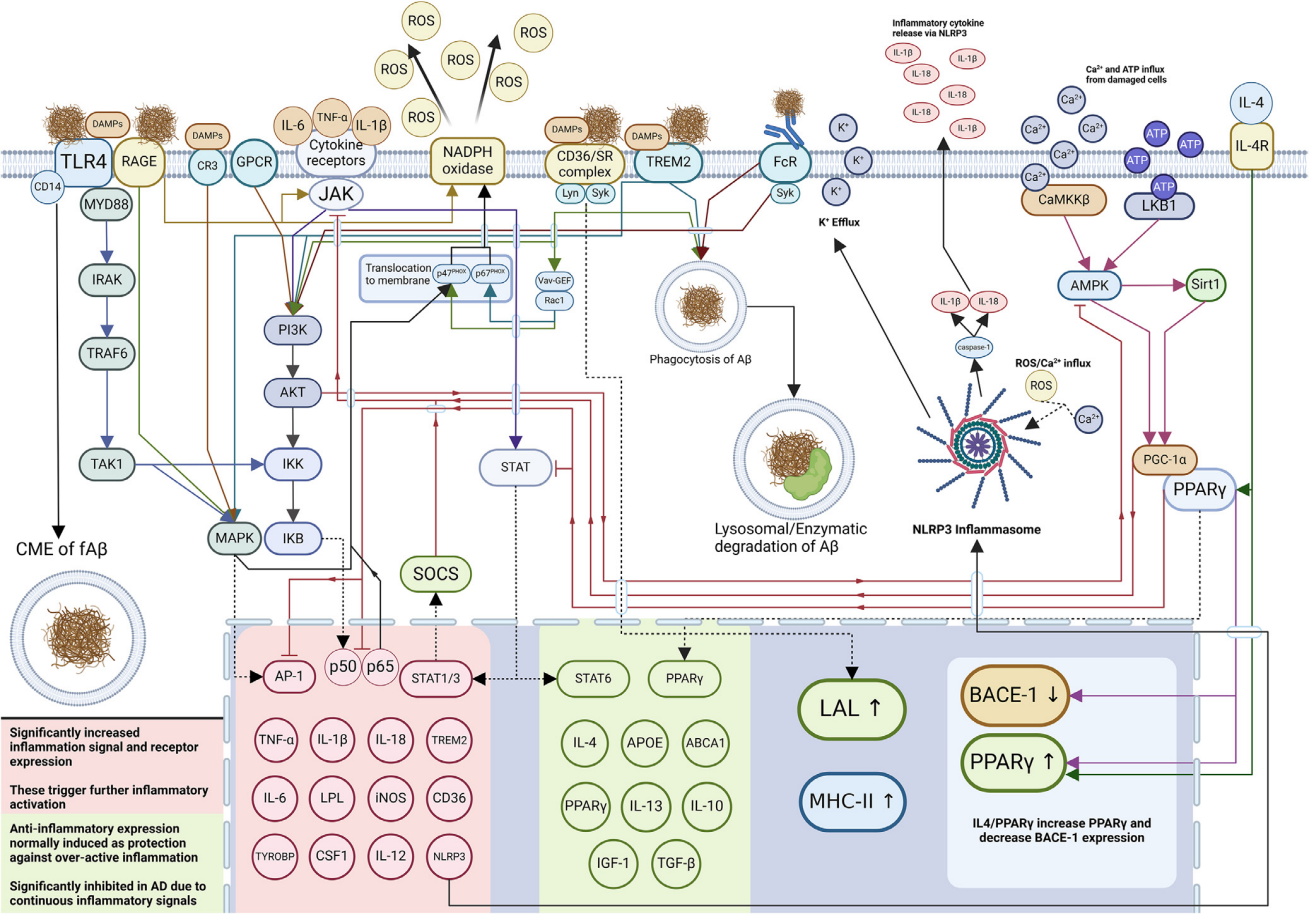
Overview of significant receptor interactions and pathways between microglia and Aβ TLR4 with CD14 is triggered by Aβ and DAMPs, causing downstream NF-κB and MAPK activation and CME of Aβ. RAGE associated with TLR4 is activated by Aβ and triggers MAPK, JAK, and NADPH oxidase. CR3 triggered by DAMPs activates MAPK. GPCR activation causes PI3K/AKT and inhibits AMPK. JAK activation triggers STAT, SOCS and PI3K/AKT. CD36/SR complexes trigger RTK induced PI3K/AKT, NADPH oxidase and uptake of Aβ/DAMPs. FcR binding triggers RTK induced PI3K/AKT and uptake of Aβ. Ca^2+^ and ATP influx trigger CaMKKβ and LKB1 which activates AMPK. AMPK activates Sirt1 and PGC-1α which activates PPARγ and inhibits JAK. PPARγ inhibits p50/p65 and AP-1 activity, increases PPARγ and decreases BACE-1 expression. IL-4 increases PPARγ expression and activity. MHC-II expression is increased in DAMs. NLRP3 expression triggers inflammasome formation, causing IL-1β and IL-18 secretion

As specialized macrophages, microglia can internalize Aβ through a suite of PRRs like TREM2, TLRs 2, 4, 6 & 9, CD14 and 36, scavenger receptors, receptor for advanced glycation end-products (RAGEs), Fc receptors, and complement receptors (Fig. 3**)**. In addition to Aβ, receptor interaction with damage-associated molecular patterns (DAMPs) is one of the key drivers of inflammation. DAMPs are signals and molecules released by damaged or apoptotic cells such as Aβ, high mobility group box 1 protein (HMGB1), cytochrome c, Ca^2+^ ions, and mitochondrial DNA (mtDNA). These DAMPs play a significant role in the activation of microglia and are abundant in the chronically damaged and inflamed state seen in AD.

#### 5.1.1 Toll-like receptors

TLRs are a family of membrane proteins highly expressed on immune cells for recognition of exogenous and endogenous pathogens. Microglia express TLR1-9 [131], with high levels of mRNA expression for TLR2, 4, 5, 7 and 9 shown in APP23 transgenic mice [132]. The roles of TLR2 and TLR4 in the uptake of Aβ are well established, having key roles in the production of inflammatory cytokines after interaction with Aβ fibrils [5,110]. Activity of TLR4 in response to fAβ requires co-stimulation with CD14 and myeloid differentiation protein 2 (MD-2), with *in vitro* studies showing significantly increased secretion of inflammatory molecules such as TNF-α, IL-6 and NO, and uptake of Aβ [133]. The inhibition of TLR4 is possible, though may cause inhibition of Aβ uptake, despite lowering inflammatory responses. A study of the TLR4 antagonist IAXO-101 demonstrated cognitive improvement, reduced Aβ volume, and lowered inflammatory responses in human *APOE-ε4^+^* female mice, though minimal effect in male mice [134], with sex-based differences possibly a result of estrogen responses.

TLR2 is also strongly implicated in the progression of AD, with microglial cells isolated from TLR2 knockdown mice showing reduced expression of proinflammatory cytokines (IL-1β, IL-6, TNF-α), iNOS, as well as CD11b, CD11c and CD68 [135]. Inhibition of TLR2 in early stage APP/PS1 mice demonstrated reduced plaque volume and cognitive improvement after 7 months of anti-TLR2 antibody treatment [136]. Overall, TLR2/4 present unique possibilities in the treatment of AD by directly affecting the clearance of Aβ and subsequent signal cascades. A window of treatment has been proposed in which inflammatory signaling may be controlled, preventing the switch of therapeutic activation to degeneration and AD progression [137].

#### 5.1.2 Estrogen

Estrogen interacts with estrogen receptors present on microglia triggering a range of anti-inflammatory responses, with ovariectomized mice showed reduced Aβ-induced neuroinflammation following β-Estradiol treatment [97,138]. AD is often associated with reduced estrogen production due to ageing. Lower estrogen levels in females and consistently low levels in males during this period, suggest a potential avenue for treatment. Studies in male rat models of AD treated with estrogen showed reduced oxidative damage and improved learning and memory function compared to untreated controls [139]. Another study demonstrated that estrogen positively regulates activity of ADAM10 – a member of the α-secretase family of enzymes – and encouraged processing of APP by ADAM10 instead of BACE-1, producing less-aggregate prone and more easily degraded soluble forms of Aβ *in vitro* and *in vivo* [140].

#### 5.1.3 CD14

CD14 is a PRR present on microglia and other macrophages and is typically involved in the uptake of LPS by TLR4, via clathrin-mediated endocytosis (CME) [141]. However, CD14^+^ microglia have demonstrated significant involvement in the clearance of Aβ [142], with knockdown of CD14 and TLR4 in MG6 microglia significantly reducing CME of fAβ [112]. There is also an age-dependent increase of CD14^+^ microglia in APdE9 mice, with the increased expression occurring around Aβ plaques and associated with increased uptake of Aβ [112]. An analysis of two cardiovascular studies, also concluded that higher levels of soluble CD14 greatly increased risk of incident dementia, and were strongly associated with accelerated brain aging, cognitive decline and neural atrophy [143].

#### 5.1.4 Scavenger receptors

Scavenger receptors (SR) are a large and structurally diverse group of cell surface receptors involved in cell adhesion and uptake of a wide array of ligands including lipoproteins, apoptotic cells, phospholipids, carbohydrates and proteoglycans [144,145]. Members of the SR family that are expressed on microglia and have interactions with Aβ include SR-A1 [146], SR-B2 (CD36) [147], SR-L1 (LRP1/low-density lipoprotein receptor-related protein 1) [148] and SR-L2 (LRP2/Megalin) [149]. SR-A1 deficiency is observed to directly increase Aβ aggregation *in vivo*, significantly increasing neurodegeneration and mortality [146], suggesting induction of SR-A1 expression may be useful as a treatment. LRP1 and 2 are used in the transport or Aβ across the BBB and out of the CNS [150]. A recent study examined the effects of LPS and all-trans retinoic acid (ATRA) on LRP2 expression in BV-2 microglia and observed significant increases in LRP2 expression and uptake of Aβ after treatment with both [149]. Additionally, ATRA may have anti-inflammatory properties by down-regulating BACE-1 expression and avoiding NO production and NF-κB activation [149,151].

#### 5.1.5 CD36

CD36 is a scavenger receptor involved in the uptake of long chain fatty acids (LCFA) and oxidized low-density lipoproteins (oxLDL), bacterial antigens [152], parasites [153] hydrophobic peptides [154] and apoptotic cell fragments [155]. Its role across these domains places it firmly in the realm of innate immunity, with downstream activation of pro-inflammatory responses well documented [156,157]. Microglia are known to express CD36 in the human CNS [158], and can effectively bind to and uptake Aβ fibrils as part of a receptor complex of CD36, CD47, SR-1A and integrin α_6_β_1_ [158,159]. This binding contributes to the characteristic swarming seen around plaques. Furthermore, this complex promotes phagocytosis, increased expression of lysosomal acid lipase (LAL), and the production of ROS like H_2_O_2_ [158,160] via stimulation of the RTKs Lyn and Syk. Aβ activates Lyn and Syk through this complex, where they then phosphorylate Vav-GEF which activates Rac1 GTPase. Activated Rac1 can then stimulate p47^PHOX^ and p67^PHOX^, triggering NADPH oxidase activity and ROS production [130]. Induction of phagocytosis and increased expression of LAL aligns with functional clearance of Aβ in the early stages of AD, with dysfunctional clearance leading to chronic inflammation from ROS [98].

#### 5.1.6 RAGE

RAGE is a member of the immunoglobulin (Ig) family of receptors, capable of binding a wide variety of ligands, including Aβ peptides, S100 proteins, HMGB1, as well as glycoproteins [101,145,[161], [162], [163]]. The activity of RAGE is multifaceted, having roles in ligand transport across the blood brain barrier (BBB) as well as signal transduction in microglia. Importantly, RAGE on microglia can activate multiple inflammatory cascades including ROS production via NADPH oxidase [164], AP-1, STAT3, and NF-κB [165]. RAGE signaling, ROS presence and NF-κB trigger a positive feedback loop that further activates the inflammatory state [[166], [167], [168], [169]]. Additionally, there is significant evidence demonstrating strong crosstalk between RAGE and TLR signals due to shared ligands, transcription factors and cascade end-products [170]. There may be therapeutic potential in the inhibition of RAGE activity. Genetic depletion of RAGE in mAPP mice demonstrated decreased Aβ load as well as reduced APP-Aβ metabolism via reduced β- and γ-secretase activity [171].

#### 5.1.7 Fc receptors

Fc receptors (FcRs) are a family of surface receptors that bind the constant (Fc) region of Igs, with specific FcRs existing for each sub-group of Ig and mediate the response of immune effector cells to antibody complexes. Microglia express Fc receptors and can bind to anti-Aβ antibodies, triggering an increase in their phagocytic activity [172,173]. However, this treatment pathway may induce a shift in microglial response towards an inflammatory state that eventually inhibits the clearance of Aβ [174]. Interestingly, anti-Aβ antibodies may promote clearance of Aβ in the CNS through “peripheral sink” activity, where antibody binding and ultimate degradation of Aβ may induce an equilibrium shift of sAβ between the CNS and periphery [175,176]. However, this theory is controversial, with other studies showing clearance of peripheral Aβ has no effect on CNS Aβ volume [177,178]. Additionally, anti-Aβ antibodies can cross the BBB in effective volumes to directly interact with microglia and CNS Aβ [179]. There is evidence showing peripheral sink activity however, as younger transgenic mice showed decreased cerebral Aβ and cognitive deficit than untreated counterparts [[180], [181], [182]], suggested to be the result of established insoluble Aβ plaques being unable to cross the BBB in the aged mice [173,175]. Regardless, clinical trials of anti-Aβ have consistently shown increased removal of plaques in treated patients, demonstrating a potential role for FcRs and antibodies in AD [[183], [184], [185]].

#### 5.1.8 Complement

The complement system is a complex chain of membrane-associated and soluble proteins interacting with the primary goal of pathogen opsonization – painting targets for phagocytic cells to recognize. The C1q dependent classical complement pathway (CCP) is a known tool of microglia and astrocytes for synaptic pruning and maintenance in the developing CNS, with C1q expression upregulated in AD pathology [186,187]. C1q can bind to Aβ, with binding efficacy most effective with Aβ_42_ and reduced in shorter peptides [188]. As C1q is elevated in AD pathologies and microglial activation is in overdrive, overzealous synaptic engulfment is common. A recent study demonstrated deletion of C1q has a protective effect, reducing synaptic loss [189]. CR3 is expressed in microglia and comprised of the subunits CD11b and CD18 and is involved in phagocytosis of synapses, Aβ, and inflammation via ROS activation [190].

### 5.2 Superoxide production

In microglia, ROS production is mediated by the NADPH oxidase subunits p47^PHOX^ and p67^PHOX^, with inflammatory signals activating translocation of these units to the cellular membrane [191,192]. The secretion of ROS has significant effects on local microglia, astrocytes, neurons, and cells of the BBB. Aβ stimulates the activation of NADPH oxidase and release of ROS through inflammatory signaling like ERK1/2 and p38 MAPK, RTK, and RAGE ligand binding [130,170]. This signals other microglia through direct interaction with ROS and DAMPs released from local cells, enabling positive regulation of inflammatory activation and further ROS release [109,191].

### 5.3 Swarming and physical barriers

Microglia are motile cells, capable of detecting pathogens or damage signals, then translocating to the site of injury/damage [193]. One of the simplest ways microglia can engage with Aβ plaques is migrating to affected areas and forming a tight physical barrier to prevent further aggregation [6]. These DAMs then phagocytose plaques, though degradation is commonly ineffective [13,109]. Inhibited breakdown of plaques contributes to inflammatory signaling that – while recruiting more microglia to the area – also further inhibits clearance and digestion of plaques, while also irritating surrounding neurons and astrocytes [194,195].

### 5.4 Proteolytic degradation

Microglia are capable of degrading soluble Aβ by secreting proteases like matrix MMP-9, IDE, and neprilysin [196,197]. This degradation functions in a dose-dependent manner, with lower volumes resulting in reduced peptide clearance [[197], [198], [199]]. Additionally, PRRs present on microglia – most notably TREM2 and CD36 – are known to directly influence phagocytosis of Aβ and Aβ-lipoprotein complexes and their subsequent lysosomal degradation [56,81,158,159].

### 5.5 Microglia as antigen-presenting cells

Microglia, as the resident macrophages in the brain, have the capability to endocytose, process, and present antigens on their surface as antigen-presenting cells (APCs) using MHC-II and co-stimulatory (CD33, -40, -80, -86) receptors. MHC-II is expressed in activated microglia, with low levels in the inactive M0 state [200]. In AD, there is conflicting evidence regarding MHC-II expression. One study noted that microglial MHC-II expression was significantly inhibited in the presence of oligomeric Aβ, impairing their antigen-presenting function in APP-PS1 AD mice [201]. These findings contrasted with an earlier study that showing that Aβ-specific Th1 cell treatment enhanced MHC-II expression in microglia and boosted their clearance activity against Aβ in the 5XFAD AD mouse model [202]. In another, it was suggested that repeated antigen-specific stimulation could lead to dysfunction in antigen-presentation, as well as increased iNOS and inflammatory signaling [203], potentially contributing to contradictory findings.

## 6 Interactions between microglia and other cells in AD pathology

**Fig. 4:**
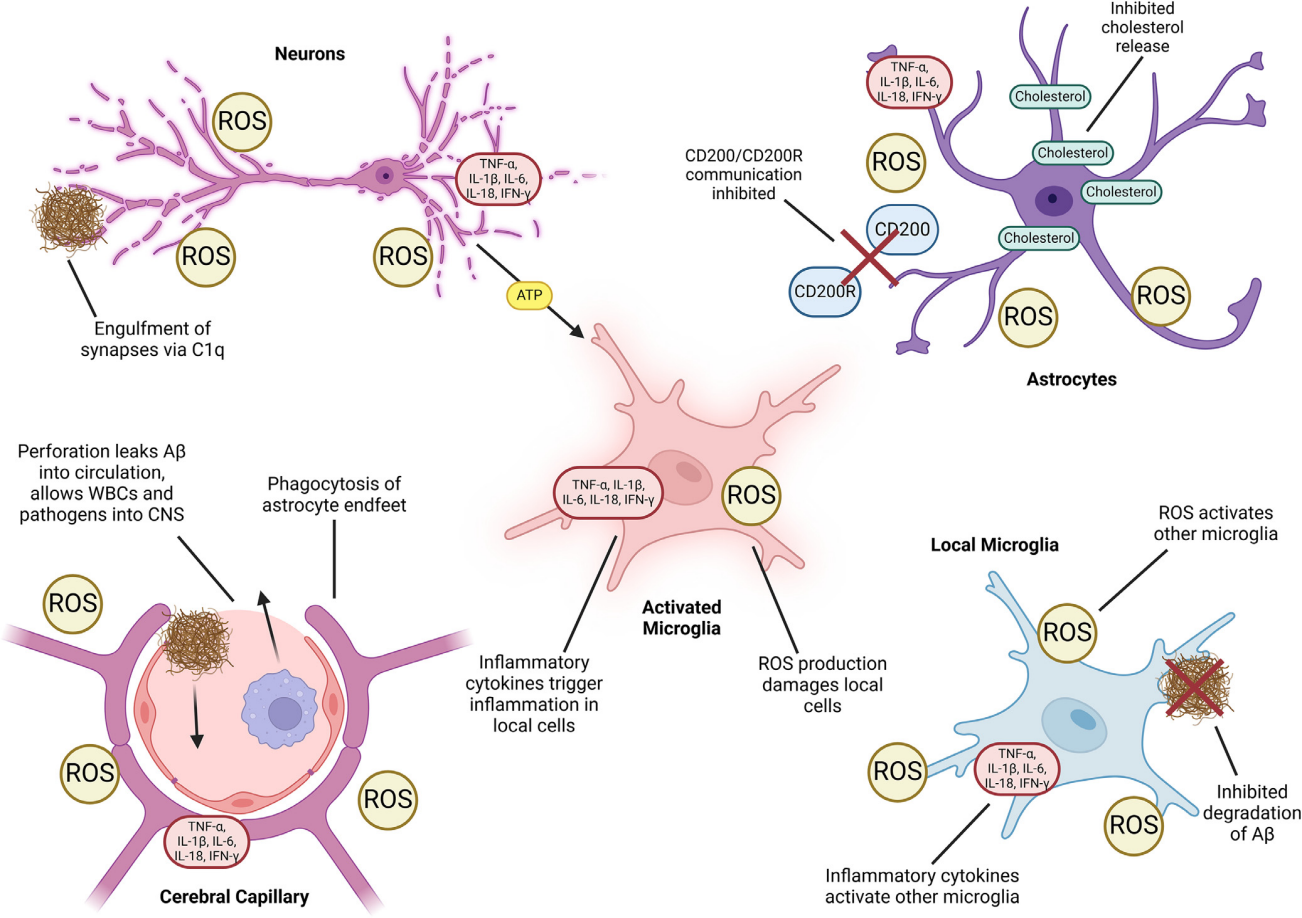
Dysfunctional interactions between activated microglia and local cells of the CNS in AD Activated microglia release inflammatory cytokines and ROS that trigger inflammation and damage in other cells. These signals trigger a chain reaction in local microglia, causing greater inflammation and diminished Aβ clearance. Microglia are unable to communicate with astrocytes via CD200/CD200R, causing unrestrained activation. Inflamed astrocytes are unable to release cholesterol to microglia, lowering microglial survival and lysosomal function. Damaged neurons experience excitotoxicity, releasing large amounts of ATP that trigger microglial activation. This activation further causes ROS production and complement signals trigger Aβ-synapse engulfment. Phagocytic microglia perforate the BBB by engulfing surrounding astrocyte end feet and damaging vascular cells, allowing Aβ efflux and influx of white blood cells, pathogens, and macromolecules.

In neurodegenerative conditions, microglia play a critical role within the CNS, yet they are just one component. In the healthy CNS, microglia communicate with other cell types to maintain CNS balance. Their surveillant behavior, facilitated by their motility, extension of processes, along with a diverse array of surface receptors, allows microglia to continuously monitor their local environment. They gather information on synaptic health, astrocyte and neuron function, local inflammatory signals, anti-inflammatory cues, and metabolic function [61]. However, in AD, this communication is disrupted (Fig. 4**)**. The typically dormant microglia convert to pro-inflammatory phenotypes and are unable to effectively send or receive the suppressive, anti-inflammatory signals that control their responses. Additionally, this lack of suppressive signaling encourages inflammatory hyperactivity, causing significant damage to themselves and other microglia, astrocytes, neurons, and local vasculature. Another facet of AD to consider is the role of the peripheral immune system. Circulating monocytes have demonstrated greater efficacy at clearing Aβ than microglia, and studies have shown this occurs within the cerebral vasculature [204]. Inhibition of infiltrated mast cells in APP/PS1 AD mice improved cognitive function and synaptic health, without affecting Aβ volume or neuroinflammation [205]. A wider discussion of the peripheral system on CNS and microglia function is beyond the scope of this review, so we recommend this review by Berriat, et al. [206] for in-depth discussion.

### 6.1 Microglia-microglia

Accurate and functional microglia-microglia communication is an important aspect of maintaining CNS homeostasis, with dysfunctional communication leading to ineffective or overactive immune responses. The initial inflammatory signals upon detection of Aβ serve to recruit other microglia in the area to the site of the plaque to either uptake and degrade it, or to swarm around it preventing its spread. However, the constant stream of inflammatory cytokines and chemokines [12], dysfunctional Aβ clearance [64], Ca^2+^ influx [116], complement signals [207], and internal signals like NF-κB [208] only trigger the further secretion of these factors.

### 6.2 Astrocytes

Communication between microglia and astrocytes is crucial in controlling the induction and reduction of immune responses within the CNS. One of the primary methods of communication between microglia and other cells of the CNS is surface-surface binding of CD200. In a physiological state, CD200 is expressed most cells of the brain, with its receptor CD200R only found on microglia and other macrophages [209]. CD200-CD200R binding is a critical mediator of microglial activation. When bound, microglia are observed in a resting and surveillant state [209]. Importantly, expression of CD200 and CD200R are significantly reduced in areas of the brain affected by AD [104,210]. This could suggest a loss of anti-inflammatory signaling between microglia and astrocytes that leads to the chronic reactive state observed in AD.

Cholesterol is another mechanism of communication between microglia and astrocytes. In the CNS, astrocytes have a significant role in cholesterol synthesis, a crucial molecule in microglial survival [211]. In cultures derived from AD brains expressing APOE-ε4, astrocytes demonstrated marked abnormal cholesterol production, increased stored lysosomal cholesterol, a significant reduction of cholesterol released to microglia, as well as significantly higher inflammatory cytokine expression [195]. Crucially, cholesterol was found to aggregate intra- and extracellularly in microglia, and can significantly inhibit its phagocytic and degradative abilities against Aβ [11]. Conversely, anti-inflammatory signals like IL-10 released from M2-like microglia can generate anti-inflammatory responses in astrocytes, triggering the secretion of TGF-β, thus reducing microglial activation [105]. Cytokine and chemokine signaling are crucial parts of microglia-astrocyte communication. Activation of microglia or astrocytes generates a signaling loop that results in the release of IL-1β and TNFα, which can further recruit microglia and astrocytes [212]. In addition, activated cells can release CCL2-5, which then bind to microglia and initiate chemotaxis, but not astrocytes [213,214].

### 6.3 Neurons

In physiological conditions, microglia contribute to neuronal and synaptic care, with functions such as neurogenesis and clearance of apoptotic neural stem cells in the developing CNS [[215], [216], [217], [218]], synaptic pruning & clearance of aberrant dendritic spines via complement cascades [187,219], and regulation of synaptic plasticity through cytokine pathways [220]. The close connection between microglia and neurons results in significant effects to neuron health in AD. Firstly, inhibited proteolysis of Aβ results in its accumulation intra- and extracellular. ATP stimulation of microglia triggers the release of internalized, intact Aβ [10]. Additionally, the detection of Aβ by microglial C3b induces inflammatory cytokine, ROS, and NO release via C5a and CD88 [190], inducing neuron membrane permeabilization, allowing influx of inflammatory signals and toxic plaques into the neuron and nucleus, causing DNA damage. Furthermore, the activity of NFTs within the neuron upregulates the expression of the GPCR adenosine A_2A_ receptor (A_2A_R), triggering the C1q complement cascade and engulfing synapses [221]. Interestingly, APOE has demonstrated high binding affinity for C1q as an inhibitor of the classical complement cascade and can be found within Aβ plaques. However, the APOE-ε4 genotype suppressed this inhibition [207].

The presence of plaques and ROS also activates the NLRP3 inflammasome in microglia, triggering lysosomal injury, the secretion of cathepsin B, IL-1β, IL-18, as well as K^+^ efflux and Ca^2+^ influx [194]. Importantly, K^+^ release further induces NLRP3 activation in a positive feedback loop similar to other inflammatory aspects of AD. This overactivation continues to secrete inflammatory factors like ROS and cytokines, irritating and damaging neurons and synapses, releasing further signals of damage, again triggering NLRP3 activation. Another factor affecting NLRP3 activation is inhibited autophagy within microglia, where impairment of this process upregulates NLRP3 expression [222]. This may also be exacerbated in patients with APOE-ε4 due to inhibition of lysosomal function [195].

### 6.4 The blood brain barrier

The BBB plays a crucial role in protecting the CNS by tightly regulating the movement of molecules into and out of the brain [223]. The BBB is comprised of astrocytes, pericytes, microglia, endothelial cells (ECs) and tight junctions, and a basement membrane, forming a tightly controlled barrier that regulates transport of molecules and cells between peripheral circulation and the brain. In AD, compromised BBB integrity significantly impairs its protective function. In AD, factors such as increased cytokines, and microglial phagocytosis of BBB-associated cells contribute to BBB damage. *In vitro* studies in mice have shown that microglia respond to EC-produced CCL-5 during inflammation, initially migrating toward cerebral vasculature to form tight junctions with ECs, which helps maintain BBB integrity [224]. However, these microglia can transition into CD68^+^ phagocytic types over time, engulfing astrocyte end-feet during chronic inflammation, further compromising BBB integrity [224]. Mitochondrial dysfunction and oxidative stress also contribute to BBB damage. Overproduction of ROS by microglia triggers inflammatory signaling and transcription factors in BBB cells, such as NF-κB and MAPK, significantly damaging mitochondrial function and respiration, especially in the mitochondria-rich ECs [223,224]. Perforation of the BBB allows for the efflux of Aβ into circulation and the cerebrospinal fluid, as well as influx of circulating macromolecules, pathogens, and white blood cells, leading to significant damage and inflammation. Additionally, BBB leakage allows for increased flow of water and Beta-like tubulins (βLTs) into the brain, leading to swelling and increased cranial pressure [225].

## 7 Therapeutic strategies targeting microglia in AD

As key instigators of the immune response in AD, immunotherapies targeting the brain inherently impact microglia. For example, monoclonal antibodies (mAbs) bind to Aβ plaques, and Fc receptors on microglia recognize these antibodies leading to the engulfment of the Aβ/mAb complex. However, modifying the activation state of microglia in AD could directly alleviate chronic neuroinflammation and neurotoxicity.

### 7.1 Immunotherapies for AD

The use of immunotherapies employs two possible approaches: active or passive. Active immunotherapies encompass the use of vaccines, either whole or fragmented pathogens that are rendered inert and administered to the patient, typically alongside an adjuvant which activates antigen presenting cells at exposure site, generating an adaptive immune response and long-term protection. Passive immunotherapies employ synthetic peptides or mAbs that are able to bind to the target pathogen for effects such as structural change and opsonization and activating immune effector cells. The lack of effective disease-modifying treatments for AD has caused a surge in research for immunotherapeutic approaches over the last 20 years, with microglia being an attractive target for intervention.

#### 7.1.1 Passive immunotherapies for AD

Recently, the US Food & Drug Administration (FDA) approved three anti-Aβ mAbs for the treatment of early AD: Aducanumab, Donanemab, and Lecanemab. The approval of Aducanumab was controversial due to ambiguous efficacy, high cost, and high adverse event incidence [183], with manufacturer Biogen discontinuing the product in 2024. These three treatments have demonstrated Aβ reduction and some effect on cognitive decline, though were associated with higher adverse events than placebo groups [184,226,227]. Administration of these treatments allows for microglial FcR binding with mAb/Aβ complexes, triggering isolation and phagocytosis of plaques, soluble oligomers and protofibrils [228,229]. Bapineuzumab, Solanezumab, Gantenerumab, and Crenezumab are single chain fragment variables (scFvs) that are similar to Aducanumab, Donanemab and Lecanemab, and underwent clinical trials, though were halted after displaying minimal effect on halting cognitive decline and Aβ deposition [185].

#### 7.1.2 Active immunotherapies for AD

While less common than passive approaches, active immunotherapies are a strong contender for anti-Aβ treatments. Vaccines induce an adaptive immune response, with one of the primary end-goals being generation of antibodies that are specific to the target pathogen. These antibodies then function similarly to mAbs, circulating until they encounter their target and binding to it, causing structural changes and activating effector cells. One study in 5XFAD mice assessed a pyroglutamate-modified Aβ species (pE_3_Aβ) vaccine, AV-1986R/A, and showed promise in rescuing cognitive function [230]. The pE_3_Aβ isoform is highly toxic and has a much higher volume and propensity to aggregation and seeding than other species [231,232], making it an attractive target for intervention. The vaccine UB-311 underwent a phase IIa trial in 2017 and demonstrated favorable safety, tolerance, and immunogenicity, but no significant effect on cognitive decline, though cognitive testing scores were low at baseline, limiting measurable effects of the treatment [233,234]. Additionally, preclinical testing of UB-311 in transgenic mice and cynomolgus macaques found no evidence of microglial activation and avoided inflammatory responses [235]. The novel vaccine CAD106 comprised of Aβ_1-6_ coupled to coat proteins of bacteriophage Qβ, and demonstrated consistent increases in anti-Aβ antibodies in APOE-ε4 homozygous patients, and no increase in Aβ deposition over the study period, but was halted early due to unfavorable results from an associated trial [236,237]. ALZ-101 is a vaccine candidate currently undergoing a phase I trial and is expected to complete in 2025 [238]. ALZ-101 is composed of stabilized Aβ_42_ oligomers that have demonstrated safety and immunogenicity in zebrafish, and mAbs derived from this vaccine – ALZ-201 – selectively target oligomeric Aβ in human AD brain tissue [239,240]. The DNA vaccine AV-1959D has shown immunogenicity and safety in mice models of AD [241,242] and is undergoing a phase I trial [243].

#### 7.1.3 The limitations of immunotherapies

The lack of significant effect on cognitive function in human trials may be due to the fact that neural tissue cannot be salvaged, repaired, or generated with our current technology and treatments. By the time symptoms of AD have presented, significant tissue loss has already occurred. Additionally, neural plasticity declines with age, significantly inhibiting the brain's ability to repair and alter pathways with remaining healthy tissue [244]. These factors hamper accurate evaluation of treatment candidates, leading to the possibility that effective treatments may currently exist, but are written-off for not affecting already damaged cognitive function. One aspect of these treatments that cannot be overlooked is the possibility of adverse events resulting from treatment. AN1792 was the first anti-Aβ vaccine and underwent a phase IIa clinical trial in 2002 [245]. While demonstrating reduction in Aβ volume, the study was halted early when approximately 6 % of participants developed meningoencephalitis following treatment. While the exact mechanism of these events is unknown, the risk of severe adverse events and reactions to treatments cannot be overlooked. Autoimmune responses, hyperactivity of effector cells, and unforeseen mechanisms are all possibilities that require thorough investigation and safety procedures.

### 7.2 Bioactive compounds that shift microglial phenotype

Activation state modulation in AD involves treating microglia with anti-inflammatory compounds that inhibit or trigger pro- and anti-inflammatory pathways like NF-κB and PPARγ (Table 1). The constant inflammatory signals expressed and received during AD keeps microglia deadlocked in the inflammatory state [12], so providing a way out of these states could reset microglia to a more stable state, capable of initiating healthy function and plaque clearance again. Many bioactive compounds exhibit anti-inflammatory effects in microglia [96,99]. Acetylcholine is able to switch M1 microglia to M2 through α7 nicotinic receptor (nAChR) activation [246]. Angiotensin II binding to the angiotensin II type I receptor (AT1) can trigger NADPH oxidase and the M1 phenotype. AT1 blockers like candesartan, by inhibiting TLR4/NF-κB pathways [208], and telmisartan, via PPARγ and CAMKKβ/AMPK pathways [116], can promote M2 polarization. Curcumin is a compound found in the turmeric plant, and exhibits anti-inflammatory effects in microglia by inhibiting TLR4/NF-κB signaling, suppressing TREM2 expression, activating CaMKKβ/AMPK signaling [99]. Additionally, Liu et al. found that curcumin as a PPARγ agonist directly inhibited M1 expression in APP/PS1 mouse models of AD, ameliorating cognitive deficit and neuroinflammation [247]. Rosmarinic acid (RA) is a polyphenol found in a large variety of herbs (i.e., rosemary, sage, mint and basil) and can shift microglia from M1 to M2 by reprogramming mitochondrial function [248]. Wei et al. found that RA could promote M2 polarization by inhibiting the phosphoinositide-dependent protein kinase 1 (PDPK1)/AKT/mTOR pathway – a downstream pathway of PI3K signaling – a decrease in hypoxia inducible factors (HIF) and phosphorylated pyruvate dehydrogenase lipoamide kinase isozyme 3 (PDK3) occurred, significantly increasing mitochondrial respiration and M2 expression [248]. Studies have demonstrated strong anti-inflammatory effects of vitamin D on microglia. Experiments showed that microglia deficient in vitamin D experienced significant reductions in inflammatory cytokine release (IL-6, IL-12, TNF-α, IFN-γ) and increases in IL-10 secretion after treatment [249,250].

**Table 1:** Compounds that have anti-inflammatory effects in microglia, their pathways, and effects on signaling and activation.

Compound	Pathway	Effect	Reference
Acetylcholine	α7 nAChR	IL-1β, IL-6 ↓ JAK2/STAT3 ↑	[246]
Anisalcohol	NF-κB, JNK	CD16/32 ↓ CD206 ↑	[251]
Astaxanthin	NF-κB, JNK	TNF-α, IL-1β ↓ CD86 ↓	[252]
ATRA	NF-κB	NF-κB ↓, BACE-1 ↓	[151]
Betulinic Acid	CaMKKβ/AMPK	AMPK ↑	[253]
Candesartan	TLR4, NF-κB	NF-κB ↓	[208]
Curcumin	TLR4, NF-κB, PPARγ	NF-κB ↓, TREM2 ↓, CaMKKβ/AMPK ↑, PPARγ ↑	[247,254]
Estrogen	NF-κB	NF-κB ↓, BACE-1 ↓, ADAM10 ↑, NEP ↑	[97,[138], [139], [140]]
Metformin	AMPK, NF-κB	AMPK ↑, NF-κB ↓	[255]
Pioglitazone	PPARγ	ABCA1 ↑, APOE ↑, IL-1β ↓, TNF-α ↓	[120,123]
Resveratrol	TLR4, NF-κB, NLRP3	TLR4 ↓, NF-κB ↓, NLRP3 ↓, IL-4 ↑	[[256], [257], [258]]
Rosiglitazone	PPARγ	JNK ↓, STAT3 ↓, PPARγ ↑	[125,259]
Rosmarinic Acid	PDPK1, AKT, mTOR	NF-κB ↓, HIF ↓, PDK3 ↓, AMPK ↑	[248]
Telmisartan	AMPK	AMPK ↑	[116]
Vitamin D	SOCS3/IL-10	IL-6 ↓, IL-12 ↓, TNF-α ↓, IFN-γ ↓, IL-10 ↑	[249,250]

### 7.3 Two treatments, one goal

Anti-inflammatory compounds alone cannot substantially modify AD. With this in mind, a two-pronged approach that utilizes anti-inflammatory treatments in conjunction with established Aβ-clearing immunotherapies may provide the answer. While further investigation is needed, inducing M2 expression may provide a “reset” to microglial activation and signaling that could enhance immunotherapy responses. Promoting the M2 state could reduce harmful effects like inflammatory transcription factor activation, inflammatory cytokine signaling, and ROS/NO production and release could repair neurotrophic functions like microglial autophagy and phagocytic ability. While *in vitro* and *in vivo* animal models have demonstrated positive anti-inflammatory effects with these treatments, there is only a small number of clinical studies on cognitive performance in AD for a small number of compounds. Curcumin is the most extensively documented, with some studies suggesting improvements in cognition [260,261], and others finding no effect [262,263]. Curcumin is also hampered by poor water solubility and low bioavailability – a challenge that is being addressed through improvements in nanotechnology. Nano formulations of curcumin (nanocurcumin) have gained significant attention in recent years and have demonstrated positive effects on neuroinflammation and immunomodulation in clinical trials for Covid-19 [264], multiple sclerosis [265], and Behcet's disease [266], though some studies show non-significant results [267]. These results suggest that nanocurcumin may have potential as a treatment, and investigations into its effects in AD may yield positive results. Resveratrol is an antioxidant typically found in grapes, berries, and peanuts that regulates TLR4 and inflammatory stimulant activity and inhibits TLR expression and the NF-κB and NLRP3 inflammasome pathways [256]. A phase II trial investigating the effects of resveratrol on neuroinflammation and adaptive immune function in AD found increases in IL-4, attenuated cognitive decline, and significantly lowered levels of CSF Aβ [258]. However, a later study observed no significant improvements to cognitive decline, contrasting with earlier results [257]. The lack of clinical data for these compounds is a significant challenge against their potential use, and continued investigation is the best pathway forward but the available data highlights that therapeutically relevant effects are present and are worth exploring.

## 8 Conclusion and future prospects

AD is highly complex, with many aspects still unclear. However, the role of microglia as drivers of chronic inflammation and their interactions with Aβ are increasingly recognized as crucial components of the disease. The intricate network of signals influencing microglial activation underscores the potential for therapies that modify their states. Traditional treatments have shown limited effectiveness, while newer immunotherapies hold promise but are still in research phases. Combining polarizing treatments with immunotherapy could potentially prevent cognitive decline more effectively by targeting plaque removal and triggering systemic repair functions. Myeloid-derived suppressor cells (MDSCs), known for suppressing immune responses, are implicated in AD and other diseases [268]. Future studies may explore how MDSCs interact with microglia, pivotal in neuroinflammation and neurodegeneration. By modulating microglial activation with anti-inflammatory cytokines, MDSCs might reduce neuroinflammation and enhance Aβ clearance. Animal models suggest increasing MDSC levels can improve cognitive function by modulating immunity. Elevated MDSC levels in AD patients may reflect a compensatory response to neuroinflammation, offering potential for therapies that modulate MDSC activity to slow disease progression. Additionally, strategies targeting microglial activation states, enhancing Aβ clearance via phagocytosis, using CRISPR for gene editing, stem cell therapies for microglial replacement, and precision targeting of microglial receptors highlight promising avenues for managing AD. Advanced technologies in bioinformatics and artificial intelligence hold promise for identifying new therapeutic targets and tailoring treatments for individual patients, potentially offering a comprehensive approach to AD management alongside existing therapies.

## Declaration of competing interest

On behalf of all authors, the corresponding author states that there is no conflict of interest.

## References

[1] Neugroschl J., Wang S. (2011). Alzheimer's disease: diagnosis and treatment across the spectrum of disease severity. Mt Sinai J Med.

[2] Nichols E., Steinmetz J.D., Vollset S.E. (2022). Estimation of the global prevalence of dementia in 2019 and forecasted prevalence in 2050: an analysis for the Global Burden of Disease Study 2019. The Lancet Public Health.

[3] Laurent C., Buee L., Blum D. (2018). Tau and neuroinflammation: What impact for Alzheimer's Disease and tauopathies?. Biomed J.

[4] Gulisano W., Maugeri D., Baltrons M.A. (2018). Role of Amyloid-β and tau proteins in Alzheimer's Disease: confuting the amyloid cascade. J Alzheimers Dis.

[5] Udan M.L.D., Ajit D., Crouse N.R., Nichols M.R. (2008). Toll-like receptors 2 and 4 mediate Aβ(1–42) activation of the innate immune response in a human monocytic cell line. J Neurochem.

[6] Zhong L., Wang Z., Wang D. (2018). Amyloid-beta modulates microglial responses by binding to the triggering receptor expressed on myeloid cells 2 (TREM2). Mol. Neurodegener..

[7] Meng J.X., Zhang Y., Saman D. (2022). Hyperphosphorylated tau self-assembles into amorphous aggregates eliciting TLR4-dependent responses. Nat Commun.

[8] Cao Q., Tan C.C., Xu W. (2020). The prevalence of dementia: a systematic review and meta-analysis. J Alzheimers Dis.

[9] Schetters S.T.T., Gomez-Nicola D., Garcia-Vallejo J.J., Van Kooyk Y. (2018). Neuroinflammation: microglia and T cells get ready to tango. Front Immunol.

[10] Badimon A., Strasburger H.J., Ayata P. (2020). Negative feedback control of neuronal activity by microglia. Nature.

[11] Garland E.F., Hartnell I.J., Boche D. (2022). Microglia and astrocyte function and communication: what do we know in humans?. Front Neurosci.

[12] Muzio L., Viotti A., Martino G. (2021). Microglia in neuroinflammation and neurodegeneration: from understanding to therapy. Front Neurosci.

[13] Keren-Shaul H., Spinrad A., Weiner A. (2017). A unique microglia type associated with restricting development of Alzheimer's Disease. Cell.

[14] Gerrits E., Brouwer N., Kooistra S.M. (2021). Distinct amyloid-β and tau-associated microglia profiles in Alzheimer's disease. Acta Neuropathol.

[15] Lal R., Lin H., Quist A.P. (2007). Amyloid beta ion channel: 3D structure and relevance to amyloid channel paradigm. Biochim Biophys Acta.

[16] Oz M., Lorke E.D., Yang S.K.-H., Petroianu G. (2013). On the interaction of β-amyloid peptides and α7-Nicotinic acetylcholine receptors in Alzheimer's Disease. Current Alzheimer Research.

[17] Swomley A.M., Butterfield D.A. (2015). Oxidative stress in Alzheimer disease and mild cognitive impairment: evidence from human data provided by redox proteomics. Arch Toxicol.

[18] Davis Parker J.William, Filley C.M., Parks J.K. (1990). Cytochrome oxidase deficiency in Alzheimer's disease. Neurology.

[19] Zhang X., Song W. (2013). The role of APP and BACE1 trafficking in APP processing and amyloid-β generation. Alzheimers Res. Ther..

[20] Capone R., Tiwari A., Hadziselimovic A. (2021). The C99 domain of the amyloid precursor protein resides in the disordered membrane phase. J Biol Chem.

[21] Querfurth H.W., LaFerla F.M. (2010). Alzheimer's Disease. N Engl J Med.

[22] Vitek M.P., Bhattacharya K., Glendening J.M. (1994). Advanced glycation end products contribute to amyloidosis in Alzheimer disease. Proc Natl Acad Sci USA.

[23] Yamazaki Y., Zhao N., Caulfield T.R., Liu C.C., Bu G. (2019). Apolipoprotein E and Alzheimer disease: pathobiology and targeting strategies. Nat Rev Neurol.

[24] Wang S., Colonna M. (2019). Microglia in Alzheimer's disease: a target for immunotherapy. J Leukoc Biol..

[25] Bateman R.J., Xiong C., Benzinger T.L. (2012). Clinical and biomarker changes in dominantly inherited Alzheimer's disease. N Engl J Med.

[26] Shankar G.M., Li S., Mehta T.H. (2008). Amyloid-beta protein dimers isolated directly from Alzheimer's brains impair synaptic plasticity and memory. Nat Med.

[27] Martin L., Latypova X., Wilson C.M. (2013). Tau protein kinases: involvement in Alzheimer's disease. Ageing Res Rev.

[28] Lindwall G., Cole R.D. (1984). Phosphorylation affects the ability of tau protein to promote microtubule assembly. J Biol Chem.

[29] Trinczek B., Ebneth A., Mandelkow E.M., Mandelkow E. (1999). Tau regulates the attachment/detachment but not the speed of motors in microtubule-dependent transport of single vesicles and organelles. J Cell Sci.

[30] Cuchillo-Ibanez I., Seereeram A., Byers H.L. (2008). Phosphorylation of tau regulates its axonal transport by controlling its binding to kinesin. FASEB J.

[31] Abasi L.S., Elathram N., Movva M., Deep A., Corbett K.D., Debelouchina G.T. (2024). Phosphorylation regulates tau's phase separation behavior and interactions with chromatin. Commun Biol.

[32] Neddens J., Temmel M., Flunkert S. (2018). Phosphorylation of different tau sites during progression of Alzheimer's disease. Acta Neuropathol Commun.

[33] Ge X., Zhang D., Qiao Y., Zhang J., Xu J., Zheng Y. (2021). Association of tau pathology with clinical symptoms in the subfields of hippocampal formation. Front. Aging Neurosci.

[34] Arriagada P.V., Growdon J.H., Hedley-Whyte E.T., Hyman B.T. (1992). Neurofibrillary tangles but not senile plaques parallel duration and severity of Alzheimer's disease. Neurology.

[35] Tissot C., Therriault J., Pascoal T.A. (2021). Association between regional tau pathology and neuropsychiatric symptoms in aging and dementia due to Alzheimer's disease, Alzheimer's & Dementia. Translational Res Clin Interv.

[36] Sengupta U., Guerrero-Muñoz M.J., Castillo-Carranza D.L. (2015). Pathological interface between oligomeric alpha-synuclein and tau in synucleinopathies. Biol Psychiatry.

[37] Hawkins B.E., Krishnamurthy S., Castillo-Carranza D.L. (2013). Rapid accumulation of endogenous tau oligomers in a rat model of traumatic brain injury: possible link between traumatic brain injury and sporadic tauopathies. J Biol Chem.

[38] Brothers H.M., Gosztyla M.L., Robinson S.R. (2018). The physiological roles of amyloid-β peptide hint at new ways to treat Alzheimer's Disease, front. Aging Neurosci.

[39] Puzzo D., Gulisano W., Arancio O., Palmeri A. (2015). The keystone of Alzheimer pathogenesis might be sought in Aβ physiology. Neuroscience.

[40] Tu S., Okamoto S., Lipton S.A., Xu H. (2014). Oligomeric Aβ-induced synaptic dysfunction in Alzheimer's disease. Mol. Neurodegener..

[41] Livingston G., Huntley J., Sommerlad A. (2020). Dementia prevention, intervention, and care: 2020 report of the Lancet Commission. Lancet North Am Ed.

[42] Booth F.W., Roberts C.K., Laye M.J. (2012). Lack of exercise is a major cause of chronic diseases. Comprehensive Physiol.

[43] Mobaderi T., Kazemnejad A., Salehi M. (2024). Exploring the impacts of risk factors on mortality patterns of global Alzheimer's disease and related dementias from 1990 to 2021. Sci Rep.

[44] Belloy M.E., Andrews S.J., Le Guen Y. (2023). APOE genotype and alzheimer disease risk across age, sex, and population ancestry. JAMA Neurol.

[45] Lim U., Wang S., Park S.Y. (2022). Risk of Alzheimer's disease and related dementia by sex and race/ethnicity: the Multiethnic Cohort Study. Alzheimers Dement.

[46] Xie J., Van Hoecke L., Vandenbroucke R.E. (2022). The impact of systemic inflammation on Alzheimer's Disease pathology. Front Immunol.

[47] Spychala M.S., Venna V.R., Jandzinski M. (2018). Age-related changes in the gut microbiota influence systemic inflammation and stroke outcome. Ann Neurol.

[48] Asarat M., Apostolopoulos V., Vasiljevic T., Donkor O. (2016). Short-chain fatty acids regulate cytokines and Th17/Treg cells in human peripheral blood mononuclear cells in vitro. Immunol Invest.

[49] Asarat M., Vasiljevic T., Apostolopoulos V., Donkor O. (2015). Short-chain fatty acids regulate secretion of IL-8 from human intestinal epithelial cell lines in vitro. Immunol Invest.

[50] Asarat M., Apostolopoulos V., Vasiljevic T., Donkor O. (2015). Short-chain fatty acids produced by synbiotic mixtures in skim milk differentially regulate proliferation and cytokine production in peripheral blood mononuclear cells. Int J Food Sci Nutr.

[51] McGarry N., Murray C.L., Garvey S. (2021). Double stranded RNA drives anti-viral innate immune responses, sickness behavior and cognitive dysfunction dependent on dsRNA length, IFNAR1 expression and age. Brain. Behav. Immun..

[52] Jurgens H.A., Amancherla K., Johnson R.W. (2012). Influenza infection induces neuroinflammation, alters hippocampal neuron morphology, and impairs cognition in adult mice. J Neurosci.

[53] Barrientos R.M., Frank M.G., Watkins L.R., Maier S.F. (2012). Aging-related changes in neuroimmune-endocrine function: implications for hippocampal-dependent cognition. Horm Behav.

[54] Feehan J., Tripodi N., Apostolopoulos V. (2021). The twilight of the immune system: The impact of immunosenescence in aging. Maturitas.

[55] Giau V.V., Bagyinszky E., Youn Y.C., An S.S.A., Kim S. (2019). APP, PSEN1, and PSEN2 mutations in asian patients with early-onset alzheimer disease. Int J Mol Sci.

[56] Zhao Y., Wu X., Li X. (2018). TREM2 Is a receptor for β-amyloid that mediates microglial function. Neuron.

[57] Castellano J.M., Kim J., Stewart F.R. (2011). Human apoE isoforms differentially regulate brain amyloid-β peptide clearance. Sci Transl Med.

[58] Sierksma A., Lu A., Mancuso R. (2020). Novel Alzheimer risk genes determine the microglia response to amyloid-β but not to TAU pathology. EMBO Mol Med.

[59] Riphagen J.M., Ramakers I.H., Freeze W.M. (2020). Linking APOE-ε4, blood-brain barrier dysfunction, and inflammation to Alzheimer's pathology. Neurobiol Aging.

[60] Rawat V., Wang S., Sima J. (2019). ApoE4 Alters ABCA1 membrane trafficking in astrocytes. J Neurosci.

[61] Butovsky O., Weiner H.L. (2018). Microglial signatures and their role in health and disease. Nat Rev Neurosci.

[62] Morrow J.A., Hatters D.M., Lu B. (2002). Apolipoprotein E4 forms a molten globule: a potential basis for its association with disease *. J Biol Chem.

[63] Li Z., Shue F., Zhao N., Shinohara M., Bu G. (2020). APOE2: protective mechanism and therapeutic implications for Alzheimer's disease. Mol. Neurodegener..

[64] Jiang Q., Lee C.Y., Mandrekar S. (2008). ApoE promotes the proteolytic degradation of Abeta. Neuron.

[65] Bosch M., Sánchez-Álvarez M., Fajardo A. (2020). Mammalian lipid droplets are innate immune hubs integrating cell metabolism and host defense. Science.

[66] Haney M.S., Pálovics R., Munson C.N. (2024). APOE4/4 is linked to damaging lipid droplets in Alzheimer's disease microglia. Nature.

[67] Kloske C.M., Dugan A.J., Weekman E.M. (2021). Inflammatory pathways are impaired in alzheimer disease and differentially associated with apolipoprotein E status. J Neuropathol Exp Neurol.

[68] Fitz N.F., Wolfe C.M., Playso B.E. (2020). Trem2 deficiency differentially affects phenotype and transcriptome of human APOE3 and APOE4 mice. Mol. Neurodegener..

[69] Fryer J.D., Simmons K., Parsadanian M. (2005). Human apolipoprotein E4 alters the amyloid-beta 40:42 ratio and promotes the formation of cerebral amyloid angiopathy in an amyloid precursor protein transgenic model. J Neurosci.

[70] Huynh T.V., Davis A.A., Ulrich J.D., Holtzman D.M. (2017). Apolipoprotein E and Alzheimer's disease: the influence of apolipoprotein E on amyloid-β and other amyloidogenic proteins. J Lipid Res.

[71] Liao F., Zhang T.J., Jiang H. (2015). Murine versus human apolipoprotein E4: differential facilitation of and co-localization in cerebral amyloid angiopathy and amyloid plaques in APP transgenic mouse models. Acta Neuropathol Commun.

[72] Halliday M.R., Rege S.V., Ma Q. (2016). Accelerated pericyte degeneration and blood-brain barrier breakdown in apolipoprotein E4 carriers with Alzheimer's disease. J Cereb Blood Flow Metab.

[73] Vance J.E., Hayashi H. (2010). Formation and function of apolipoprotein E-containing lipoproteins in the nervous system. Biochim Biophys Acta.

[74] Tang W., Tam J.H., Seah C. (2015). Arf6 controls beta-amyloid production by regulating macropinocytosis of the Amyloid Precursor Protein to lysosomes. Mol. Brain.

[75] Mukhamedova N., Hoang A., Cui H.L. (2016). Small GTPase ARF6 regulates endocytic pathway leading to degradation of ATP-binding cassette transporter A1. Arterioscler Thromb Vasc Biol.

[76] Hirsch-Reinshagen V., Zhou S., Burgess B.L. (2004). Deficiency of ABCA1 impairs apolipoprotein E metabolism in brain. J Biol Chem.

[77] Koldamova R., Staufenbiel M., Lefterov I. (2005). Lack of ABCA1 considerably decreases brain ApoE level and increases amyloid deposition in APP23 mice. J Biol Chem.

[78] Deczkowska A., Weiner A., Amit I. (2020). The physiology, pathology, and potential therapeutic applications of the TREM2 signaling pathway. Cell.

[79] Yuan P., Condello C., Keene C.D. (2016). TREM2 haplodeficiency in mice and humans impairs the microglia barrier function leading to decreased amyloid compaction and severe axonal dystrophy. Neuron.

[80] Wang Y., Ulland T.K., Ulrich J.D. (2016). TREM2-mediated early microglial response limits diffusion and toxicity of amyloid plaques. J Exp Med.

[81] Wang Y., Cella M., Mallinson K. (2015). TREM2 lipid sensing sustains the microglial response in an Alzheimer's Disease model. Cell.

[82] Colonna M. (2023). The biology of TREM receptors. Nat Rev Immunol.

[83] Sudom A., Talreja S., Danao J. (2018). Molecular basis for the loss-of-function effects of the Alzheimer's disease-associated R47H variant of the immune receptor TREM2. J Biol Chem.

[84] Song W.M., Joshita S., Zhou Y., Ulland T.K., Gilfillan S., Colonna M. (2018). Humanized TREM2 mice reveal microglia-intrinsic and -extrinsic effects of R47H polymorphism. J Exp Med.

[85] Turner P.R., O'Connor K., Tate W.P., Abraham W.C. (2003). Roles of amyloid precursor protein and its fragments in regulating neural activity, plasticity and memory. Prog Neurobiol.

[86] Priller C., Bauer T., Mitteregger G., Krebs B., Kretzschmar H.A., Herms J. (2006). Synapse formation and function is modulated by the amyloid precursor protein. J Neurosci.

[87] Moir R.D., Lathe R., Tanzi R.E. (2018). The antimicrobial protection hypothesis of Alzheimer's disease. Alzheimer's & Dementia.

[88] Duce J.A., Tsatsanis A., Cater M.A. (2010). Iron-export ferroxidase activity of β-amyloid precursor protein is inhibited by zinc in Alzheimer's disease. Cell.

[89] Mann D.M.A., Davidson Y.S., Robinson A.C. (2018). Patterns and severity of vascular amyloid in Alzheimer's disease associated with duplications and missense mutations in APP gene, Down syndrome and sporadic Alzheimer's disease. Acta Neuropathol.

[90] Lu D.C., Rabizadeh S., Chandra S. (2000). A second cytotoxic proteolytic peptide derived from amyloid β-protein precursor. Nat Med.

[91] Park S.A., Shaked G.M., Bredesen D.E., Koo E.H. (2009). Mechanism of cytotoxicity mediated by the C31 fragment of the amyloid precursor protein. Biochem Biophys Res Commun.

[92] Lu D.C., Soriano S., Bredesen D.E., Koo E.H. (2003). Caspase cleavage of the amyloid precursor protein modulates amyloid β-protein toxicity. J Neurochem.

[93] Orobets K.S., Karamyshev A.L. (2023). Amyloid precursor protein and Alzheimer's Disease. Int J Mol Sci.

[94] Szaruga M., Munteanu B., Lismont S. (2017). Alzheimer's-causing mutations shift Aβ length by destabilizing γ-Secretase-Aβn Interactions. Cell.

[95] Sarasija S., Laboy J.T., Ashkavand Z., Bonner J., Tang Y., Norman K.R. (2018). Presenilin mutations deregulate mitochondrial Ca(2+) homeostasis and metabolic activity causing neurodegeneration in Caenorhabditis elegans. eLife.

[96] Darwish S.F., Elbadry A.M.M., Elbokhomy A.S., Salama G.A., Salama R.M. (2023). The dual face of microglia (M1/M2) as a potential target in the protective effect of nutraceuticals against neurodegenerative diseases. Front Aging.

[97] Yun J., Yeo I.J., Hwang C.J. (2018). Estrogen deficiency exacerbates Aβ-induced memory impairment through enhancement of neuroinflammation, amyloidogenesis and NF-ĸB activation in ovariectomized mice. Brain Behav Immun.

[98] Subhramanyam C.S., Wang C., Hu Q., Dheen S.T. (2019). Microglia-mediated neuroinflammation in neurodegenerative diseases. Semin Cell Dev Biol.

[99] Guo S., Wang H., Yin Y. (2022). Microglia polarization from M1 to M2 in neurodegenerative diseases. Front. Aging Neurosci.

[100] Zhang G., Wang Z., Hu H., Zhao M., Sun L. (2021). Microglia in Alzheimer's disease: a target for therapeutic intervention. Front. Cell. Neurosci..

[101] Fang F., Lue L.F., Yan S. (2010). RAGE-dependent signaling in microglia contributes to neuroinflammation, Abeta accumulation, and impaired learning/memory in a mouse model of Alzheimer's disease. FASEB J.

[102] Krasemann S., Madore C., Cialic R. (2017). The TREM2-APOE pathway drives the transcriptional phenotype of dysfunctional microglia in neurodegenerative diseases. Immunity.

[103] Hickman S.E., Allison E.K., El Khoury J. (2008). Microglial dysfunction and defective beta-amyloid clearance pathways in aging Alzheimer's disease mice. J Neurosci.

[104] Walker D.G., Dalsing-Hernandez J.E., Campbell N.A., Lue L.F. (2009). Decreased expression of CD200 and CD200 receptor in Alzheimer's disease: a potential mechanism leading to chronic inflammation. Exp Neurol.

[105] Norden D.M., Fenn A.M., Dugan A., Godbout J.P. (2014). TGFβ produced by IL-10 redirected astrocytes attenuates microglial activation. Glia.

[106] Yin Z., Rosenzweig N., Kleemann K.L. (2023). APOE4 impairs the microglial response in Alzheimer's disease by inducing TGFβ-mediated checkpoints. Nat Immunol.

[107] Liu C.-C., Wang N., Chen Y. (2023). Cell-autonomous effects of APOE4 in restricting microglial response in brain homeostasis and Alzheimer's disease. Nat Immunol.

[108] Sun N., Victor M.B., Park Y.P. (2023). Human microglial state dynamics in Alzheimer's disease progression. Cell.

[109] Jorfi M., Maaser-Hecker A., Tanzi R.E. (2023). The neuroimmune axis of Alzheimer's disease. Genome Med.

[110] Reed-Geaghan E.G., Savage J.C., Hise A.G., Landreth G.E. (2009). CD14 and toll-like receptors 2 and 4 are required for fibrillar Aβ-stimulated microglial activation. The Journal of Neuroscience.

[111] Kawai T., Akira S. (2007). Signaling to NF-kappaB by Toll-like receptors. Trends Mol Med.

[112] Fujikura M., Iwahara N., Hisahara S. (2019). CD14 and toll-like receptor 4 promote fibrillar Aβ42 uptake by microglia through a clathrin-mediated pathway. J Alzheimers Dis.

[113] Zhang H., Li Y., Yu J. (2013). Rho kinase inhibitor fasudil regulates microglia polarization and function. NeuroImmunoModulation.

[114] Zhang B., Wei Y.Z., Wang G.Q., Li D.D., Shi J.S., Zhang F. (2018). Targeting MAPK pathways by naringenin modulates microglia M1/M2 polarization in lipopolysaccharide-stimulated cultures. Front. Cell. Neurosci.

[115] Jeon S.M. (2016). Regulation and function of AMPK in physiology and diseases. Exp Mol Med.

[116] Wang Y., Huang Y., Xu Y. (2018). A Dual AMPK/Nrf2 activator reduces brain inflammation after stroke by enhancing microglia M2 polarization. Antioxid Redox Signal.

[117] Marcelo K.L., Means A.R., York B. (2016). The Ca(2+)/Calmodulin/CaMKK2 Axis: Nature's Metabolic CaMshaft. Trends Endocrinol Metab.

[118] Cantó C., Auwerx J. (2009). PGC-1alpha, SIRT1 and AMPK, an energy sensing network that controls energy expenditure. Curr Opin Lipidol.

[119] Hernandez-Quiles M., Broekema M.F., Kalkhoven E. (2021). PPARgamma in metabolism, immunity, and cancer: unified and diverse mechanisms of action. Front. Endocrinol. (Lausanne).

[120] Heneka M.T., Sastre M., Dumitrescu-Ozimek L. (2005). Acute treatment with the PPARgamma agonist pioglitazone and ibuprofen reduces glial inflammation and Abeta1-42 levels in APPV717I transgenic mice. Brain.

[121] Colonna M., Butovsky O. (2017). Microglia function in the central nervous system during health and neurodegeneration. Annu Rev Immunol.

[122] Jacobi D., Stanya K.J., Lee C.H. (2012). Adipose tissue signaling by nuclear receptors in metabolic complications of obesity. Adipocyte.

[123] Mandrekar-Colucci S., Karlo J.C., Landreth G.E. (2012). Mechanisms underlying the rapid peroxisome proliferator-activated receptor-γ-mediated amyloid clearance and reversal of cognitive deficits in a murine model of Alzheimer's disease. J Neurosci.

[124] Mandrekar-Colucci S., Landreth G.E. (2011). Nuclear receptors as therapeutic targets for Alzheimer's disease. Expert Opin Ther Targets.

[125] Khan M.A., Alam Q., Haque A. (2019). Current progress on peroxisome proliferator-activated receptor gamma agonist as an emerging therapeutic approach for the treatment of Alzheimer's Disease: an update. Curr Neuropharmacol.

[126] Xin P., Xu X., Deng C. (2020). The role of JAK/STAT signaling pathway and its inhibitors in diseases. Int Immunopharmacol.

[127] Gao Q., Liang X., Shaikh A.S., Zang J., Xu W., Zhang Y. (2018). JAK/STAT signal transduction: promising attractive targets for immune, inflammatory and hematopoietic diseases. Curr Drug Targets.

[128] Johnstone M., Bennett N., Standifer C. (2017). Characterization of the pro-inflammatory cytokine IL-1β on butyrate oxidation in colorectal cancer cells. J Cell Biochem.

[129] Rangarajan P., Karthikeyan A., Dheen S.T. (2016). Role of dietary phenols in mitigating microglia-mediated neuroinflammation. Neuromolecular Med.

[130] Wilkinson B., Koenigsknecht-Talboo J., Grommes C., Lee C.Y.D., Landreth G. (2006). Fibrillar beta-amyloid-stimulated intracellular signaling cascades require Vav for induction of respiratory burst and phagocytosis in monocytes and microglia. J Biol Chem.

[131] Olson J.K., Miller S.D. (2004). Microglia initiate central nervous system innate and adaptive immune responses through multiple TLRs. J Immunol.

[132] Frank S., Copanaki E., Burbach G.J., Müller U.C., Deller T. (2009). Differential regulation of toll-like receptor mRNAs in amyloid plaque-associated brain tissue of aged APP23 transgenic mice. Neurosci Lett.

[133] Walter S., Letiembre M., Liu Y. (2007). Role of the toll-like receptor 4 in neuroinflammation in Alzheimer's disease. Cell Physiol Biochem.

[134] Balu D., Valencia-Olvera A.C., Nguyen A. (2023). A small-molecule TLR4 antagonist reduced neuroinflammation in female E4FAD mice. Alzheimers Res. Ther..

[135] Jana M., Palencia C.A., Pahan K. (2008). Fibrillar amyloid-beta peptides activate microglia via TLR2: implications for Alzheimer's disease. J Immunol.

[136] McDonald C.L., Hennessy E., Rubio-Araiz A. (2016). Inhibiting TLR2 activation attenuates amyloid accumulation and glial activation in a mouse model of Alzheimer's disease. Brain Behav Immun.

[137] Dallas M.L., Widera D. (2021). TLR2 and TLR4-mediated inflammation in Alzheimer's disease: self-defense or sabotage?. Neural Regen Res.

[138] Acosta-Martínez M. (2020). Shaping microglial phenotypes through estrogen receptors: relevance to sex-specific neuroinflammatory responses to brain injury and disease. J Pharmacol Exp Ther.

[139] Pan Q., Guo K., Li Y., Tu Q. (2019). [Role of TXNIP-mediated oxidative stress in delaying Alzheimer's disease by estrogen]. Zhong Nan Da Xue Xue Bao Yi Xue Ban.

[140] Kim J.Y., Mo H., Kim J. (2022). Mitigating effect of estrogen in Alzheimer's Disease-mimicking cerebral organoid. Front Neurosci.

[141] Husebye H., Halaas Ø., Stenmark H. (2006). Endocytic pathways regulate Toll-like receptor 4 signaling and link innate and adaptive immunity. EMBO J.

[142] Liu Y., Walter S., Stagi M. (2005). LPS receptor (CD14): a receptor for phagocytosis of Alzheimer's amyloid peptide. Brain.

[143] Pase M.P., Himali J.J., Beiser A.S. (2020). Association of CD14 with incident dementia and markers of brain aging and injury. Neurology.

[144] Zani I.A., Stephen S.L., Mughal N.A. (2015). Scavenger receptor structure and function in health and disease. Cells.

[145] Apostolopoulos V., Thalhammer T., Tzakos A.G., Stojanovska L. (2013). Targeting antigens to dendritic cell receptors for vaccine development. J Drug Deliv.

[146] Frenkel D., Wilkinson K., Zhao L. (2013). Scara1 deficiency impairs clearance of soluble amyloid-β by mononuclear phagocytes and accelerates Alzheimer's-like disease progression. Nat Commun.

[147] Means T.K., Mylonakis E., Tampakakis E. (2009). Evolutionarily conserved recognition and innate immunity to fungal pathogens by the scavenger receptors SCARF1 and CD36. J Exp Med.

[148] Kanekiyo T., Bu G. (2014). The low-density lipoprotein receptor-related protein 1 and amyloid-β clearance in Alzheimer's disease, Front. Aging Neurosci.

[149] Takano-Kawabe K., Matoba K., Nakamura Y., Moriyama M. (2024). Low Density Lipoprotein Receptor-related Protein 2 expression and function in cultured astrocytes and microglia. Neurochem Res.

[150] Zlokovic B.V. (2004). Clearing amyloid through the blood–brain barrier. J Neurochem.

[151] Wang R., Chen S., Liu Y. (2015). All-trans retinoic acid reduces BACE1 expression under inflammatory conditions via modulation of nuclear factor kappa B (NF-κB) Signaling. J Biol Chem.

[152] Truong-Bolduc Q.C., Khan N.S., Vyas J.M., Hooper D.C. (2017). Tet38 efflux pump affects staphylococcus aureus internalization by epithelial cells through interaction with CD36 and contributes to bacterial escape from acidic and nonacidic phagolysosomes. Infect Immun.

[153] Thylur R.P., Wu X., Gowda N.M. (2017). CD36 receptor regulates malaria-induced immune responses primarily at early blood stage infection contributing to parasitemia control and resistance to mortality. J Biol Chem.

[154] Abumrad N.A., Ajmal M., Pothakos K., Robinson J.K. (2005). CD36 expression and brain function: does CD36 deficiency impact learning ability?. Prostaglandins Other Lipid Mediat.

[155] Penberthy K.K., Ravichandran K.S. (2016). Apoptotic cell recognition receptors and scavenger receptors. Immunol Rev.

[156] Castleman M.J., Febbraio M., Hall P.R. (2015). CD36 Is essential for regulation of the host innate response to staphylococcus aureus α-toxin–mediated dermonecrosis. J Immunol.

[157] Sheedy F.J., Grebe A., Rayner K.J. (2013). CD36 coordinates NLRP3 inflammasome activation by facilitating intracellular nucleation of soluble ligands into particulate ligands in sterile inflammation. Nat Immunol.

[158] Coraci I.S., Husemann J., Berman J.W. (2002). CD36, a class B scavenger receptor, is expressed on microglia in Alzheimer's Disease brains and can mediate production of reactive oxygen species in response to β-amyloid fibrils. Am J Pathol.

[159] Stewart C.R., Stuart L.M., Wilkinson K. (2010). CD36 ligands promote sterile inflammation through assembly of a Toll-like receptor 4 and 6 heterodimer. Nat Immunol.

[160] Woo M.-S., Yang J., Beltran C., Cho S. (2016). Cell surface CD36 protein in monocyte/macrophage contributes to phagocytosis during the resolution phase of ischemic stroke in mice*. J Biol Chem.

[161] Akirav E.M., Preston-Hurlburt P., Garyu J. (2012). RAGE expression in human T cells: a link between environmental factors and adaptive immune responses. PLoS One.

[162] Leclerc E., Fritz G., Vetter S.W., Heizmann C.W. (2009). Binding of S100 proteins to RAGE: an update. Biochim Biophys Acta.

[163] Neeper M., Schmidt A.M., Brett J. (1992). Cloning and expression of a cell surface receptor for advanced glycosylation end products of proteins. J Biol Chem.

[164] Wautier M.-P., Chappey O., Corda S., Stern D.M., Schmidt A.M., Wautier J.-L. (2001). Activation of NADPH oxidase by AGE links oxidant stress to altered gene expression via RAGE. Am J Physiol Endocrinol Metab.

[165] Rojas A., Delgado-López F., González I., Pérez-Castro R., Romero J., Rojas I. (2013). The receptor for advanced glycation end-products: A complex signaling scenario for a promiscuous receptor. Cell. Signal..

[166] Bierhaus A., Schiekofer S., Schwaninger M. (2001). Diabetes-associated sustained activation of the transcription factor nuclear factor-κB. Diabetes.

[167] Rojas A., Figueroa H., Morales E. (2009). Fueling inflammation at tumor microenvironment: the role of multiligand/rage axis. Carcinogenesis.

[168] Rojas A., Mercadal E., Figueroa H., Morales A.M. (2008). Advanced glycation and ROS: a link between diabetes and heart failure. Curr Vasc Pharmacol.

[169] Rojas A., Morales M.A. (2004). Advanced glycation and endothelial functions: A link towards vascular complications in diabetes. Life Sci.

[170] Gąsiorowski K., Brokos B., Echeverria V., Barreto G.E., Leszek J. (2018). RAGE-TLR crosstalk sustains chronic inflammation in neurodegeneration. Mol Neurobiol.

[171] Fang F., Yu Q., Arancio O. (2018). RAGE mediates Aβ accumulation in a mouse model of Alzheimer's disease via modulation of β- and γ-secretase activity. Hum Mol Genet.

[172] Fuller J.P., Stavenhagen J.B., Teeling J.L. (2014). New roles for Fc receptors in neurodegeneration-the impact on Immunotherapy for Alzheimer's Disease. Front Neurosci.

[173] Wilcock D.M., Munireddy S.K., Rosenthal A., Ugen K.E., Gordon M.N., Morgan D. (2004). Microglial activation facilitates Abeta plaque removal following intracranial anti-Abeta antibody administration. Neurobiol Dis.

[174] Wilcock D.M., Zhao Q., Morgan D. (2011). Diverse inflammatory responses in transgenic mouse models of Alzheimer's disease and the effect of immunotherapy on these responses. ASN Neuro.

[175] DeMattos R.B., Bales K.R., Cummins D.J., Paul S.M., Holtzman D.M. (2002). Brain to plasma amyloid-beta efflux: a measure of brain amyloid burden in a mouse model of Alzheimer's disease. Science.

[176] Lee V.M. (2001). Abeta immunization: moving Abeta peptide from brain to blood. Proc Natl Acad Sci USA.

[177] Georgievska B., Gustavsson S., Lundkvist J. (2015). Revisiting the peripheral sink hypothesis: inhibiting BACE1 activity in the periphery does not alter β-amyloid levels in the CNS. J Neurochem.

[178] Henderson S.J., Andersson C., Narwal R. (2014). Sustained peripheral depletion of amyloid-β with a novel form of neprilysin does not affect central levels of amyloid-β. Brain.

[179] Pardridge W.M. (2020). Treatment of Alzheimer's Disease and blood-brain barrier drug delivery. Pharmaceuticals (Basel).

[180] Janus C., Pearson J., McLaurin J. (2000). A beta peptide immunization reduces behavioural impairment and plaques in a model of Alzheimer's disease. Nature.

[181] Morgan D., Diamond D.M., Gottschall P.E. (2000). A beta peptide vaccination prevents memory loss in an animal model of Alzheimer's disease. Nature.

[182] Schenk D., Barbour R., Dunn W. (1999). Immunization with amyloid-β attenuates Alzheimer-disease-like pathology in the PDAPP mouse. Nature.

[183] Wu W., Ji Y., Wang Z. (2023). The FDA-approved anti-amyloid-β monoclonal antibodies for the treatment of Alzheimer's disease: a systematic review and meta-analysis of randomized controlled trials. Eur J Med Res.

[184] van Dyck C.H., Swanson C.J., Aisen P. (2023). Lecanemab in Early Alzheimer's Disease. N. Engl. J. Med.

[185] Valiukas Z., Ephraim R., Tangalakis K., Davidson M., Apostolopoulos V., Feehan J. (2022). Immunotherapies for Alzheimer's Disease-A Review. Vaccines (Basel).

[186] Bohlen C.J., Friedman B.A., Dejanovic B., Sheng M. (2019). Microglia in brain development, homeostasis, and neurodegeneration. Annu Rev Genet.

[187] Schafer D.P., Lehrman E.K., Kautzman A.G. (2012). Microglia sculpt postnatal neural circuits in an activity and complement-dependent manner. Neuron.

[188] Jiang H., Burdick D., Glabe C.G., Cotman C.W., Tenner A.J. (1994). beta-Amyloid activates complement by binding to a specific region of the collagen-like domain of the C1q A chain. J Immunol.

[189] Dejanovic B., Wu T., Tsai M.-C. (2022). Complement C1q-dependent excitatory and inhibitory synapse elimination by astrocytes and microglia in Alzheimer's disease mouse models. Nature Aging.

[190] Zhang J., Malik A., Choi Hyun B., Ko Rebecca W.Y., Dissing-Olesen L., MacVicar Brian A. (2014). Microglial CR3 activation triggers long-term synaptic depression in the hippocampus via NADPH oxidase. Neuron.

[191] Shimohama S., Tanino H., Kawakami N. (2000). Activation of NADPH oxidase in Alzheimer's Disease brains. Biochem Biophys Res Commun.

[192] Sheng K.-C., Pietersz G.A., Tang C.K., Ramsland P.A., Apostolopoulos V. (2010). Reactive oxygen species level defines two functionally distinctive stages of inflammatory dendritic cell development from mouse bone marrow. J Immunol.

[193] Hanisch U.-K., Kettenmann H. (2007). Microglia: active sensor and versatile effector cells in the normal and pathologic brain. Nat Neurosci.

[194] Wu A.-G., Zhou X.-G., Qiao G. (2021). Targeting microglial autophagic degradation in NLRP3 inflammasome-mediated neurodegenerative diseases. Ageing Res Rev.

[195] Tcw J., Qian L., Pipalia N.H. (2022). Cholesterol and matrisome pathways dysregulated in astrocytes and microglia. Cell.

[196] King J.V., Liang W.G., Scherpelz K.P., Schilling A.B., Meredith S.C., Tang W.J. (2014). Molecular basis of substrate recognition and degradation by human presequence protease. Structure.

[197] Shen Y., Joachimiak A., Rosner M.R., Tang W.J. (2006). Structures of human insulin-degrading enzyme reveal a new substrate recognition mechanism. Nature.

[198] Iwata N., Tsubuki S., Takaki Y. (2001). Metabolic regulation of brain Abeta by neprilysin. Science.

[199] Zipfel P., Rochais C., Baranger K., Rivera S., Dallemagne P. (2020). Matrix metalloproteinases as New Targets in Alzheimer's Disease: opportunities and challenges. J Med Chem.

[200] Jurga A.M., Paleczna M., Kuter K.Z. (2020). Overview of general and discriminating markers of differential microglia phenotypes. Front. Cell. Neurosci..

[201] Gericke C., Mallone A., Engelhardt B., Nitsch R.M., Ferretti M.T. (2020). Oligomeric forms of human amyloid-Beta(1-42) inhibit antigen presentation. Front Immunol.

[202] Mittal K., Eremenko E., Berner O. (2019). CD4 T Cells induce A subset of MHCII-expressing microglia that attenuates alzheimer pathology. iScience.

[203] Prasad S., Singh A., Hu S., Sheng W.S., Chauhan P., Lokensgard J.R. (2023). Dysregulated brain regulatory T cells fail to control reactive gliosis following repeated antigen stimulation. iScience.

[204] Michaud J.P., Bellavance M.A., Préfontaine P., Rivest S. (2013). Real-time in vivo imaging reveals the ability of monocytes to clear vascular amyloid beta. Cell Rep.

[205] Li T., Martin E., Abada Y.S. (2020). Effects of chronic masitinib treatment in APPswe/PSEN1dE9 transgenic mice modeling Alzheimer's Disease. J Alzheimers Dis.

[206] Berriat F., Lobsiger C.S., Boillée S. (2023). The contribution of the peripheral immune system to neurodegeneration. Nat Neurosci.

[207] Yin C., Ackermann S., Ma Z. (2019). ApoE attenuates unresolvable inflammation by complex formation with activated C1q. Nat Med.

[208] Qie S., Ran Y., Lu X. (2020). Candesartan modulates microglia activation and polarization via NF-κB signaling pathway. Int J Immunopathol Pharmacol.

[209] Hernangómez M., Mestre L., Correa F.G. (2012). CD200-CD200R1 interaction contributes to neuroprotective effects of anandamide on experimentally induced inflammation. Glia.

[210] Koning N., Swaab D.F., Hoek R.M., Huitinga I. (2009). Distribution of the immune inhibitory molecules CD200 and CD200R in the normal central nervous system and multiple sclerosis lesions suggests neuron-glia and glia-glia interactions. J Neuropathol Exp Neurol.

[211] Goshi N., Morgan R.K., Lein P.J., Seker E. (2020). A primary neural cell culture model to study neuron, astrocyte, and microglia interactions in neuroinflammation. J. Neuroinflammation.

[212] Choi S.S., Lee H.J., Lim I., Satoh J., Kim S.U. (2014). Human astrocytes: secretome profiles of cytokines and chemokines. PLoS One.

[213] Hu S., Chao C.C., Ehrlich L.C. (1999). Inhibition of microglial cell RANTES production by IL-10 and TGF-beta. J. Leukoc. Biol..

[214] Peterson P.K., Hu S., Salak-Johnson J., Molitor T.W., Chao C.C. (1997). Differential production of and migratory response to beta chemokines by human microglia and astrocytes. J Infect Dis.

[215] Arnò B., Grassivaro F., Rossi C. (2014). Neural progenitor cells orchestrate microglia migration and positioning into the developing cortex. Nat Commun.

[216] Squarzoni P., Thion M.S., Garel S. (2015). Neuronal and microglial regulators of cortical wiring: usual and novel guideposts. Front Neurosci.

[217] Luo C., Koyama R., Ikegaya Y. (2016). Microglia engulf viable newborn cells in the epileptic dentate gyrus. Glia.

[218] Li Y., Du X.F., Du J.L. (2013). Resting microglia respond to and regulate neuronal activity in vivo. Commun. Integr. Biol..

[219] Stevens B., Allen N.J., Vazquez L.E. (2007). The classical complement cascade mediates CNS synapse elimination. Cell.

[220] Vezzani A., Viviani B. (2015). Neuromodulatory properties of inflammatory cytokines and their impact on neuronal excitability. Neuropharmacology.

[221] Dejanovic B., Huntley M.A., De Mazière A. (2018). Changes in the synaptic proteome in tauopathy and rescue of tau-induced synapse loss by C1q antibodies. Neuron.

[222] Houtman J., Freitag K., Gimber N., Schmoranzer J., Heppner F.L., Jendrach M. (2019). Beclin1-driven autophagy modulates the inflammatory response of microglia via NLRP3. EMBO J.

[223] Song K., Li Y., Zhang H. (2020). Oxidative stress-mediated blood-brain barrier (BBB) disruption in neurological diseases. Oxid. Med. Cell. Longev..

[224] Haruwaka K., Ikegami A., Tachibana Y. (2019). Dual microglia effects on blood brain barrier permeability induced by systemic inflammation. Nat Commun.

[225] Alahmari A. (2021). Blood-brain barrier overview: structural and functional correlation. Neural Plast.

[226] Swanson C.J., Zhang Y., Dhadda S. (2021). A randomized, double-blind, phase 2b proof-of-concept clinical trial in early Alzheimer's disease with lecanemab, an anti-Aβ protofibril antibody. Alzheimers Res. Ther..

[227] Sims J.R., Zimmer J.A., Evans C.D. (2023). Donanemab in early symptomatic Alzheimer Disease: The TRAILBLAZER-ALZ 2 randomized clinical trial. JAMA.

[228] Sevigny J., Chiao P., Bussière T. (2016). The antibody aducanumab reduces Aβ plaques in Alzheimer's disease. Nature.

[229] Vitek G.E., Decourt B., Sabbagh M.N. (2023). Lecanemab (BAN2401): an anti-beta-amyloid monoclonal antibody for the treatment of Alzheimer disease. Expert Opin Investig Drugs.

[230] Zagorski K., King O., Hovakimyan A. (2023). Novel vaccine against pathological pyroglutamate-modified amyloid beta for prevention of Alzheimer's Disease. Int J Mol Sci.

[231] Liu K., Solano I., Mann D. (2006). Characterization of Abeta11-40/42 peptide deposition in Alzheimer's disease and young Down's syndrome brains: implication of N-terminally truncated Abeta species in the pathogenesis of Alzheimer's disease. Acta Neuropathol.

[232] Portelius E., Lashley T., Westerlund A. (2015). Brain amyloid-beta fragment signatures in pathological ageing and Alzheimer's disease by hybrid immunoprecipitation mass spectrometry. Neurodegener. Dis..

[233] Yu H.J., Dickson S.P., Wang P.-N. (2023). Safety, tolerability, immunogenicity, and efficacy of UB-311 in participants with mild Alzheimer's disease: a randomised, double-blind, placebo-controlled, phase 2a study. eBioMedicine.

[234] A Randomized, Double-blind, Placebo-controlled, 3-arm Parallel-group, Multicenter, Phase IIa Study to Evaluate the Safety, Tolerability, Immunogenicity, and Efficacy of UBITh® AD Immunotherapeutic Vaccine (UB-311) in Patients With Mild Alzheimer's Disease, 2015.

[235] Wang C.Y., Wang P.N., Chiu M.J. (2017). UB-311, a novel UBITh(®) amyloid β peptide vaccine for mild Alzheimer's disease. Alzheimers Dement (N Y).

[236] Riviere M.E., Langbaum J.B., Turner R.S. (2024). Effects of the active amyloid beta immunotherapy CAD106 on PET measurements of amyloid plaque deposition in cognitively unimpaired APOE ε4 homozygotes. Alzheimers Dement.

[237] A Randomized, Double-blind, Placebo-controlled, Two-cohort, Parallel Group Study to Evaluate the Efficacy of CAD106 and CNP520 in Participants at Risk for the Onset of Clinical Symptoms of Alzheimer's Disease, in: I. Banner Alzheimer's, A. National Institute on, Amgen (Eds.) 2015.

[238] A Double-blind, Randomized, Parallel-group multiple dose study on the safety, tolerability and immunogenicity of ALZ-101 in participants with early Alzheimer's Disease, in: C. Oy (Ed.) 2022.

[239] Sandberg A., Berenjeno E., Rodriguez R.Crespo (2022). Aβ42 oligomer-specific antibody ALZ-201 reduces the neurotoxicity of Alzheimer's disease brain extracts. Alzheimers Res. Ther..

[240] Sandberg A., Rodriguez R.C., Kettunen P. (2020). Specific targeting of a highly toxic subpopulation of Aβ42 oligomers for the treatment of Alzheimer's disease. Alzheimer's & Dementia.

[241] Davtyan H., Hovakimyan A., Kiani Shabestari S. (2019). Testing a MultiTEP-based combination vaccine to reduce Aβ and tau pathology in Tau22/5xFAD bigenic mice. Alzheimers Res. Ther..

[242] Petrushina I., Hovakimyan A., Harahap-Carrillo I.S. (2020). Characterization and preclinical evaluation of the cGMP grade DNA based vaccine, AV-1959D to enter the first-in-human clinical trials. Neurobiol Dis.

[243] Phase A, Randomized I (2022). Patients With Early Alzheimer's Disease.

[244] Magee J.C., Grienberger C. (2020). Synaptic plasticity forms and functions. Annu. Rev. Neurosci.

[245] Gilman S., Koller M., Black R.S. (2005). Clinical effects of Abeta immunization (AN1792) in patients with AD in an interrupted trial. Neurology.

[246] Zhang Q., Lu Y., Bian H., Guo L., Zhu H. (2017). Activation of the α7 nicotinic receptor promotes lipopolysaccharide-induced conversion of M1 microglia to M2. Am J Transl Res.

[247] Liu Z.J., Li Z.H., Liu L. (2016). Curcumin attenuates beta-amyloid-induced neuroinflammation via activation of peroxisome proliferator-activated receptor-gamma function in a rat model of Alzheimer's Disease. Front Pharmacol.

[248] Wei Y., Chen J., Cai G.-E. (2021). Rosmarinic Acid Regulates Microglial M1/M2 Polarization via the PDPK1/Akt/HIF Pathway Under Conditions of Neuroinflammation. Inflammation.

[249] Cui P., Lu W., Wang J. (2023). Microglia/macrophages require vitamin D signaling to restrain neuroinflammation and brain injury in a murine ischemic stroke model. J. Neuroinflammation.

[250] Boontanrart M., Hall S.D., Spanier J.A., Hayes C.E., Olson J.K. (2016). Vitamin D3 alters microglia immune activation by an IL-10 dependent SOCS3 mechanism. J Neuroimmunol.

[251] Xiang B., Xiao C., Shen T., Li X. (2018). Anti-inflammatory effects of anisalcohol on lipopolysaccharide-stimulated BV2 microglia via selective modulation of microglia polarization and down-regulation of NF-κB p65 and JNK activation. Mol Immunol.

[252] Wen X., Xiao L., Zhong Z. (2017). Astaxanthin acts via LRP-1 to inhibit inflammation and reverse lipopolysaccharide-induced M1/M2 polarization of microglial cells. Oncotarget.

[253] Li C., Zhang C., Zhou H. (2018). Inhibitory effects of betulinic acid on LPS-Induced Neuroinflammation Involve M2 Microglial Polarization via CaMKKβ-Dependent AMPK activation. Front. Mol. Neurosci..

[254] Small G.W., Siddarth P., Li Z. (2018). Memory and brain amyloid and tau effects of a bioavailable form of curcumin in non-demented adults: a double-blind, placebo-controlled 18-month trial. Am J Geriatr Psychiatry.

[255] Abdi M., Pasbakhsh P., Shabani M. (2021). Metformin therapy attenuates pro-inflammatory microglia by inhibiting NF-κB in cuprizone demyelinating mouse model of multiple sclerosis. Neurotox Res.

[256] Moudgil K.D., Venkatesha S.H. (2022). The anti-inflammatory and immunomodulatory activities of natural products to control autoimmune inflammation. Int J Mol Sci.

[257] Huhn S., Beyer F., Zhang R. (2018). Effects of resveratrol on memory performance, hippocampus connectivity and microstructure in older adults - A randomized controlled trial. Neuroimage.

[258] Moussa C., Hebron M., Huang X. (2017). Resveratrol regulates neuro-inflammation and induces adaptive immunity in Alzheimer's disease. J. Neuroinflammation.

[259] Chao C., Li Y., Li Q., Wu G. (2023). Inhibitory effect and mechanism of Rosiglitazone on M1 type polarization of central microglia in intracerebral hemorrhage mice based on JNK/STAT3 signaling pathway. Brain and Behavior.

[260] Small G.W., Siddarth P., Li Z. (2018). Memory and brain amyloid and tau effects of a bioavailable form of curcumin in non-demented adults: a double-blind, placebo-controlled 18-month trial. Am J Geriatr Psychiatry.

[261] Rainey-Smith S.R., Brown B.M., Sohrabi H.R. (2016). Curcumin and cognition: a randomised, placebo-controlled, double-blind study of community-dwelling older adults. Br J Nutr.

[262] Ringman J.M., Frautschy S.A., Teng E. (2012). Oral curcumin for Alzheimer's disease: tolerability and efficacy in a 24-week randomized, double blind, placebo-controlled study. Alzheimers Res. Ther..

[263] Baum L., Lam C.W., Cheung S.K. (2008). Six-month randomized, placebo-controlled, double-blind, pilot clinical trial of curcumin in patients with Alzheimer disease. J Clin Psychopharmacol.

[264] Ahmadi R., Salari S., Sharifi M.D. (2021). Oral nano-curcumin formulation efficacy in the management of mild to moderate outpatient COVID-19: A randomized triple-blind placebo-controlled clinical trial. Food Sci Nutr.

[265] Dolati S., Ahmadi M., Aghebti-Maleki L. (2018). Nanocurcumin is a potential novel therapy for multiple sclerosis by influencing inflammatory mediators. Pharmacol. Rep..

[266] Farzaneh R., Khabbazi A., Soltani-Zangbar M.S. (2022). Effects of nanocurcumin supplementation on T-helper 17 cells inflammatory response in patients with Behcet's disease: a randomized controlled trial. Immunopharmacol Immunotoxicol.

[267] Hassanizadeh S., Shojaei M., Bagherniya M., Orekhov A.N., Sahebkar A. (2023). Effect of nano-curcumin on various diseases: a comprehensive review of clinical trials. Biofactors.

[268] Kong Y.Y., Fuchsberger M., Xiang S.D., Apostolopoulos V., Plebanski M. (2013). Myeloid derived suppressor cells and their role in diseases. Curr Med Chem.

